# Multigene Phylogeny, Beauvericin Production and Bioactive Potential of *Fusarium* Strains Isolated in India

**DOI:** 10.3390/jof8070662

**Published:** 2022-06-24

**Authors:** Shiwali Rana, Sanjay Kumar Singh, Laurent Dufossé

**Affiliations:** 1 National Fungal Culture Collection of India, Biodiversity and Palaeobiology Group, MACS’ Agharkar Research Institute, G.G. Agarkar Road, Pune 411004, India; shiwalirana@aripune.org; 2 Faculty of Science, Savitribai Phule Pune University, Ganeshkhind Road, Ganeshkhind, Pune 411007, India; 3 Chembiopro Chimie et Biotechnologie des Produits Naturels, ESIROI Département Agroalimentaire, Université de la Réunion, F-97490 Sainte-Clotilde, Ile de La Réunion, France

**Keywords:** antimicrobial, fermentation, *Fusarium*, multilocus DNA sequencing, synergistic effect

## Abstract

The taxonomy of the genus *Fusarium* has been in a *flux* because of ambiguous circumscription of species-level identification based on morphotaxonomic criteria. In this study, multigene phylogeny was conducted to resolve the evolutionary relationships of 88 Indian *Fusarium* isolates based on the internal transcribed spacer region, 28S large subunit, translation elongation factor 1-alpha, RNA polymerase second largest subunit, beta-tubulin and calmodulin gene regions. *Fusarium* species are well known to produce metabolites such as beauvericin (BEA) and enniatins. These identified isolates were subjected to fermentation in *Fusarium*-defined media for BEA production and tested using TLC, HPLC and HRMS. Among 88 isolates studied, 50 were capable of producing BEA, which varied from 0.01 to 15.82 mg/g of biomass. *Fusarium tardicrescens* NFCCI 5201 showed maximum BEA production (15.82 mg/g of biomass). The extract of *F*. *tardicrescens* NFCCI 5201 showed promising antibacterial activity against *Staphylococcus aureus* MLS16 MTCC 2940 and *Micrococcus luteus* MTCC 2470 with MIC of 62.5 and 15.63 µg/mL, respectively. Similarly, the *F*. *tardicrescens* NFCCI 5201 extract in potato dextrose agar (40 µg/mL) exhibited antifungal activity in the food poison technique against plant pathogenic and other fungi, *Rhizoctonia solani* NFCCI 4327, *Sclerotium rolfsii* NFCCI 4263, *Geotrichum candidum* NFCCI 3744 and *Pythium* sp. NFCCI 3482, showing % inhibition of 84.31, 49.76, 38.22 and 35.13, respectively. The antibiotic effect was found to synergize when *Fusarium* extract and amphotericin B (20 µg/mL each in potato dextrose agar) were used in combination against *Rhizopus* sp. NFCCI 2108, *Sclerotium rolfsii* NFCCI 4263, *Bipolaris sorokiniana* NFCCI 4690 and *Absidia* sp. NFCCI 2716, showing % inhibition of 50.35, 79.37, 48.07 and 76.72, respectively. The extract also showed satisfactory dose-dependent DPPH radical scavenging activity with an IC_50_ value of 0.675 mg/mL. This study reveals the correct identity of the Indian *Fusarium* isolates based on multigene phylogeny and also throws light on BEA production potential, suggesting their possible applicability in the medicine, agriculture and industry.

## 1. Introduction

Members of the genus *Fusarium* are ubiquitous and found in soil, air and on plants [1]. *Fusarium* is an important group of plant-pathogenic fungi which affect a vast diversity of crops in all climatic zones across the globe [2]. Being a genus of great concern, accurate and correct identification of its species becomes a necessity. However, its taxonomy has been in flux due to ambiguous circumscription based on morphotaxonomic criteria. Over the past two decades, many phylogenetic studies have established that morphological species recognition frequently fails to distinguish many *Fusarium* species [3]. Phylogenetic studies have revealed that nuclear ribosomal DNA (nrDNA) is nearly useless for species-level recognition in the case of *Fusarium* and related genera; these markers are useful only in the discrimination of *Fusarium* species complexes [4]. Discordance on using the nuclear rDNA internal transcribed spacer 2 (ITS2) gene region is because of the highly divergent ITS2 rDNA xenologs or paralogs discovered in many species’ complexes [5,6]. Many protein-coding genes have been explored for identification and taxonomic purposes in *Fusarium* and related genera. The two primary genes commonly used nowadays for identification that offer high discriminatory power and are well represented in public databases are translation elongation factor 1-alpha (*tef-1α*) and RNA polymerase second largest subunit (*rpb2*). *Tef1-α* is the most common marker, which has very good resolution power for most species [7], and *Rpb2* provides enhanced discrimination between closely related species [8,9,10]; however, PCR amplification and sequencing success rate is better for *Tef1-α* than for *Rpb2*. In multigene phylogenetic analyses, additional genetic markers are often used with the previously mentioned genes, including *β-tub*, *CaM* and *Rpb1*; these markers vary in resolution depending on species complexes [11].

*Fusarium* spp. are known to produce several secondary metabolites including beauvericin (BEA) and enniatins. BEA is a cyclic hexadepsipeptide that consists of alternating D-2-hydroxyisovaleric acid and N-methylphenylalanine [12]. BEA is a potent bioactive compound that exhibits very effective anticancer, cytotoxic, antiplatelet aggregation, antimicrobial, leishmanicidal and insecticidal activities [13]. These activities are primarily due to its ionophoric properties that generally disrupt the normal physiological concentration of cations across the plasma membrane [14,15,16]. It releases Ca^2+^ from internal Ca^2+^ stores of the endoplasmic reticulum [17].

Studies prove that BEA can exhibit promising antibacterial activity against gram-positive or gram-negative bacteria, for example, *Agrobacterium tumefaciens*, *Bacillus cereus*, *B. pumilus*, *B. sphaericus*, *B. subtilis*, *B. mycoides*, *Bifidobacterium adolescentis*, *Clostridium perfringens*, *Escherichia coli*, *Eubacterium biforme*, *Mycobacterium tuberculosis*, *Paenibacillus azotofixans*, *P. alvei*, *P. pulvifaciens*, *P. validus P. macquariensis*, *Peptostreptococcus productus*, *P. anaerobius*, *Pseudomonas lachrymans*, *Salmonella typhimurium*, *Staphylococcus haemolyticus*, *Vibrio fisheri* and *Xanthomonas vesicatoria* are inhibited by BEA [18,19,20,21,22,23].

There has been public concern about certain opportunistic fungi that can cause infections; particularly, cases of *Candida albicans* are greater than before. Patients with compromised immune systems, such as those receiving organ transplants and cancer chemotherapy or those infected by human immunodeficiency virus (HIV), are more prone to such opportunistic infections [24]. BEA alone does not exhibit any antifungal activity; however, it has been found to enhance miconazole activity against *C. albicans* (wild and fluconazole-resistant) [25]. The activity of azole antifungals becomes enhanced against azole-resistant *Candida* isolates by BEA via inhibition of multidrug efflux [26]. The BEA with ketoconazole is known to synergize the antifungal effect against *Candida albicans* and *C. tropicalis*, signifying BEA’s possible use as a co-drug for antifungal infections [23,27,28]. A promising strategy using BEA, which involves simultaneous targeting drug resistance and morphogenesis, can boost antifungal efficacy against human fungal pathogens to combat life-threatening fungal infections.

In this study, 88 isolates of *Fusarium* and related genera from various substrates were identified by multigene phylogeny based on the internal transcribed spacer region, translation elongation factor 1-alpha, 28S large subunit, RNA polymerase second largest subunit, beta-tubulin and calmodulin gene regions. Further, these isolates were screened for beauvericin production. The isolate which showed maximum BEA production was subjected to large-scale fermentation, and its extract was used to study antibacterial, antifungal and antioxidant potential.

## 2. Materials and Methods

### 2.1. Isolates and Fungarium Specimens

First, 64 fungal strains identified as *Fusarium* based on morphology were procured from the National Fungal Culture Collection of India (NFCCI-WDCM 932) Table 1. The bacterial isolates used in this study, *Escherichia coli* MTCC 739, *Bacillus subtilis* MTCC 121, *Micrococcus luteus* MTCC 2470, *Raoultella planticola* MTCC 530, *Pseudomonas aeruginosa* MTCC 2453, *Staphylococcus aureus* MLS16 MTCC 2940 and *Staphylococcus aureus* MTCC 96, were procured from Microbial Type Culture Collection (MTCC). Additionally, fungal pathogens *Pythium* sp. NFCCI 3482, *Geotrichum candidum* NFCCI 3744, *Rhizoctonia solani* NFCCI 4327, *Rhizopus* sp. NFCCI 2108, *Sclerotium rolfsii* NFCCI 4263, *Bipolaris sorokiniana* NFCCI 4690 and *Absidia* sp. NFCCI 2716 were also procured from the National Fungal Culture Collection of India (NFCCI) to study the antifungal activity.

### 2.2. Collection, Isolation and Morphological Identification

During the course of this study, *Fusarium* and other related fungi were isolated from various substrates collected from different regions of India. Table 2 enlists the 24 isolates which were isolated afresh in this study along with details of their host, place of collection, date of collection and date of isolation. Pure cultures of all 24 isolates were raised using a single spore isolation technique.

**Figure 1:**
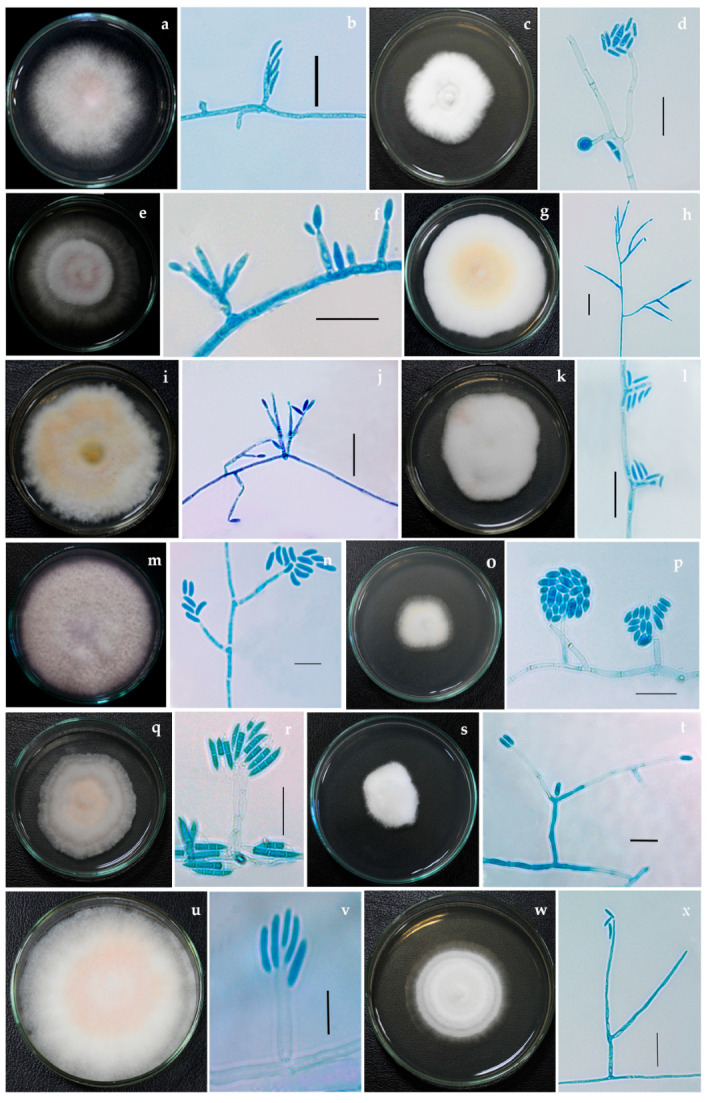
*Fusarium* isolates with phialides bearing conidia (**a**,**b**) *Fusarium nirenbergiae* NFCCI 5189 (**c**,**d**) *Neocosmospora solani* NFCCI 2315 (**e**,**f**) *F. mangiferae* NFCCI 2885 (**g**,**h**) *F. annulatum* NFCCI 2962 (**i**,**j**) *F. microconidium* NFCCI 3020 (**k**,**l**) *F. sacchari* NFCCI 3147 (**m**,**n**) *N. oblonga* NFCCI 2150 (**o**,**p**) *Albonectria rigidiuscula* NFCCI 5202 (**q**,**r**) *F. irregulare* NFCCI 2460 (**s**,**t**) *Neocosmospora suttoniana* NFCCI 2961 (**u**,**v**) *F. annulatum* NFCCI 2964 (**w**,**x**) *Neocosmospora vasinfecta* NFCCI 2972. Scale bars: b, d, f, h, j, l, p, r, t, v, x = 20 μm; n = 10 μm.

**Figure 2:**
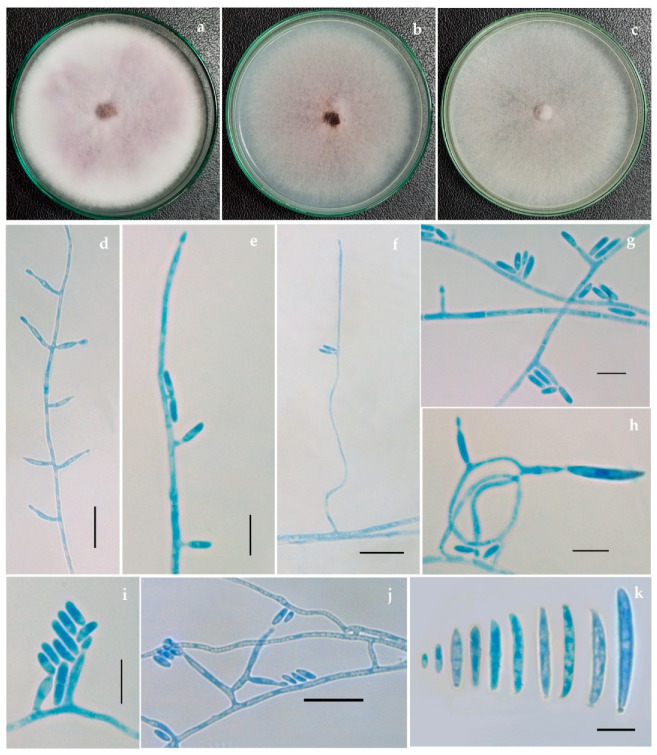
*Fusarium tardicrescens* (NFCCI 5201) culture grown on (**a**) Potato dextrose agar (**b**) Malt extract agar (**c**) Cornmeal agar (**d**–**j**) Phialides arising from mycelial hyphae producing micro and macroconidia (**k**) Micro and macroconidia. Scale bars: d, f, j = 20 μm; e, g, h, i, k = 10 μm.

Colony characteristics of all *Fusarium* isolates were studied on multiple media, such as potato dextrose agar (PDA), malt extract agar (MEA), potato carrot agar (PCA), oatmeal agar (OMA), cornmeal agar (CMA) and synthetic nutrient agar (SNA). Microscopic structures of these isolates were observed using lactophenol-cotton blue under a Carl Zeiss Image Analyzer 2 (Germany) microscope. Measurements and photomicrographs of the fungal structures were recorded using Axiovision Rel 4.8 software and Digi-Cam with an attached Carl Zeiss Image Analyzer 2 microscope (Figure 1 and Figure 2). The pure cultures are deposited and accessioned in the National Fungal Culture Collection of India (NFCCI 5189-5212) (Table 2).

### 2.3. DNA Extraction, PCR Amplification and DNA Sequencing

Genomic DNA was isolated from pure colonies of all 88 isolates growing on Potato Dextrose Agar (PDA) petri-plates, incubated for a week’s time using a simple and rapid DNA extraction protocol using FastPrep^®^24 tissue homogenizer (MP Biomedicals GmbH, Eschwege, Germany) [29]. Partial gene sequences of isolates were determined for six gene markers, i.e., ITS, *tef1-α*, LSU, *rpb2*, *β-tub* and *CaM*. The primer sets used to amplify particular gene region are summarized in Table 3.

PCR was carried out in a 25 μL reaction using 12.5 μL 2X Invitrogen Platinum SuperFi PCR Mastermix, 2 μL template DNA (10–20 ng), 1.5 μL 10 pmol primer, 5 μL 5X GC enhancer and H_2_O (Sterile Ultra-Pure Water, Sigma, St. Louis, MO, USA), with the volume made to 25 μL. The conditions of the thermo-cycling involved:

An initial denaturation at 94 °C for 5 min, 35 cycles of 1 min at 94 °C, 30 s at 52 °C, 1 min at 72 °C and a final extension at 72 °C for 8 min for ITS gene region;

An initial denaturation of 5 min at 94 °C, 30 cycles of 45 s at 94 °C, 30 s at 57 °C and 1 min at 72 °C followed by a final 7 min extension at 72 °C for *Tef1-α*;

5 min denaturation at 94 °C, 35 cycles of 1 min at 94 °C, 50 s at 52 °C and 1.2 min at 72 °C with a final 8 min extension at 72 °C for LSU;

5 min denaturation at 95 °C, 35 cycles of 45 s at 95 °C, 1 min at 52 °C and 1.5 min at 72 °C with a final 10 min extension at 72 °C for *Rpb2*;

2 min denaturation at 94 °C, 40 cycles of 35 s at 94 °C, 55 s at 52 °C and 2 min at 72 °C with a final 10 min extension at 72 °C for *β-tub*;

An initial denaturation of 5 min at 94 °C, 30 cycles of 1 min at 94 °C, 30 s at 55 °C and 2 min at 72 °C followed by a final 7 min extension at 72 °C for *CaM*.

The PCR amplicons were purified with a FavorPrep™ PCR purification kit as per the manufacturer’s instructions. Purified PCR products of all marker genes were checked on 1.2% agarose electrophoresis gels stained with ethidium bromide and were further subjected to a sequencing PCR reaction using a BigDye^®^Terminator v3.1 Cycle Sequencing Kit, as per manufacturer’s instructions.

The sequencing PCR reaction of 20 µL included 4 µL of 5× sequencing buffer, 2 µL of BigDye™ Terminator premix, 4 µL of primer (5 pmol), 4 µL of the purified amplicon and H_2_O (Sterile Ultra-Pure Water, Sigma), with the volume made to 20 μL. Thermal cycling conditions consisted of an initial denaturing at 96 °C for 3 min, followed by 30 cycles of 94 °C for 10 s, 50 °C for 40 s and 60 °C for 4 min. The BigDye^®^ terminators and salts were removed from using The BigDye Xterminator^®^ Purification Kit (Thermo Fisher Scientific, Waltham, MA, USA) as per the manufacturer’s instructions. The purified sequencing products were transferred into a 96-well microplate. The sequence was elucidated on Applied Biosystems SeqStudio Genetic Analyzer (Applied Biosystems, Foster City, CA, USA). Sequences obtained were submitted in the NCBI GenBank (accession numbers in Table S1).

### 2.4. Phylogenetic Analysis

To determine the phylogenetic status of all 88 isolates, the Internal transcribed spacer region of the nrDNA (ITS), translation elongation factor 1-alpha (*tef1-α*), 28S large subunit of the nrDNA (LSU), RNA polymerase second largest subunit (*rpb2*), beta-tubulin (*β-tub*) and calmodulin (*CaM*) loci were used to compare the isolates used in this study with already known authentic strains in the genus *Fusarium.* The sequences of the type/reference strains were retrieved from NCBI. A total of 186 isolates of *Fusarium* and related genera were used in the phylogenetic analysis (Table S1). *Atractium stilbaster* CBS 410.67 was selected as the outgroup taxon. The chosen strains used in the construction of the phylogenetic tree, along with their accession numbers and other related details, are listed in Table S1. Each gene region was individually aligned using MAFFT v. 6.864b [37]. The alignments were checked and adjusted manually using Aliview [38]. Further, alignments were concatenated and subjected to phylogenetic analyses. The best substitution model out of 286 DNA models was figured using ModelFinder [39]. Additionally, windows version IQ-tree tool v.1.6.11 [40] was used to construct the phylogenetic tree. The reliability of the tree branches was assessed and tested based on 1000 ultrafast bootstrap (UFBoot) support replicates and a SH-like approximate likelihood ratio test (SH-like aLRT) with 1000 replicates. The constructed phylogenetic tree was visualized in FigTree v.1.4.4 (http://tree.bio.ed.ac.uk/software/figtree/, accessed on 20 February 2022).

### 2.5. Fermentation for Beauvericin Production

*Fusarium* isolates were subcultured onto PDA and incubated at 25 °C for a week. Once the cultures were mature enough, spore suspensions were prepared separately for all isolates. Then, 10 µL of Tween 20 was added to 10 mL of normal saline (0.89%) and mixed adequately, saline solution was added to the culture and the spores were harvested carefully with the help of a sterile cotton swab and vortexed. The spore suspension’s optical density (OD) was adjusted between 0.08 and 0.1 at 530 nm [41]. Next, 1 mL of this spore suspension was added to 100 mL of *Fusarium* defined media (FDM) (Sucrose 25 g, Sodium nitrate (NaNO_3_) 4.25 g, Sodium chloride (NaCl) 5 g, Magnesium sulfate heptahydrate (MgSO_4_·7H_2_O) 2.5 g, Potassium dihydrogen phosphate (KH_2_PO_4_) 1.36 g, Ferrous sulphate heptahydrate (FeSO_4_·7H_2_O) 0.01 g, Zinc sulfate heptahydrate (ZnSO_4_·7H_2_O) 0.0029 g/L; pH 5.5) [42] in a 250 mL Erlenmeyer flask and was incubated in a shaking incubator at 25 °C at 150 rpm for 7 days (Figure S1).

### 2.6. Extraction of Beauvericin

Beauvericin was extracted from the fermented broth as per the protocol described earlier, with slight modification [43]. The fermented broth was filtered using Whatman filter paper no. 4. Biomass was collected, washed twice with distilled water and was allowed to dry at 50 °C. Dried biomass was extracted overnight in 25 mL of acetonitrile:water 90:10 (*v*/*v*). Further, the mixture was sonicated twice for 15 min. The mixture was filtered; filtrate was extracted with 25 mL of heptane and separated using a separating funnel. The bottom layer was evaporated to dryness in a rotary evaporator (Heidolph, Schwabach, Germany). The dry residue was dissolved in 25 mL of methanol:water, 60:40 (*v*/*v*) and extracted twice with 25 mL of dichloromethane. The dichloromethane phase containing beauvericin (BEA) was collected and evaporated to dryness in a rotary evaporator. The evaporated extract containing beauvericin was dissolved in 1 mL of Acetonitrile.

### 2.7. Detection of Beauvericin

The presence of beauvericin was initially qualitatively analyzed by thin-layer chromatography (TLC) and quantitatively by high-performance liquid chromatography (HPLC). In addition, high-resolution mass spectrometry (HRMS) was carried out for a few samples for further confirmation.

#### 2.7.1. Thin-Layer Chromatography (TLC)

About 5 to 10 µL of the extracts were spotted onto TLC Silica gel plates (TLC Silica gel 60 F254; Merck life science Pvt. Ltd., Bengaluru, Karnataka, India) along with standard Beauvericin (BEA) (500 mg/mL) (Sigma) with the help of glass capillary tube. TLC plates were developed in petroleum ether:ethyl acetate (1:1) as a solvent system. The plates were air-dried, and consequently, the spots formed on the TLC plates were detected using iodine vapours. Retention factor (Rf) values for the standard and extracts were measured as described earlier [44,45]. The extracts were further subjected to high-performance liquid chromatography (HPLC).

#### 2.7.2. High-Performance Liquid Chromatography (HPLC)

The amount of BEA in the extracts was determined by HPLC (Waters Corporation, Milford, MA, USA) using a C18 column and acetonitrile: H_2_O (85:15 *v*/*v*) as the mobile phase at a flow rate of 1 mL/min under isocratic conditions and U.V. detection at 210 nm [46]. A stock solution of standard beauvericin with a concentration of 1 mg/mL was prepared in acetonitrile and was further diluted to 15.6 µg/mL, 31.25 µg/mL, 62.5 µg/mL, 125 µg/mL, 250 µg/mL and 500 µg/mL. The retention time for BEA was found to be 9.1 min. The injection volume was 20 µL. The peak area was calibrated to the amount of BEA standard (Sigma). The amount of BEA produced was calculated with respect to the area under the peak.

#### 2.7.3. High-Resolution Mass Spectrometry (HRMS)

The mass of beauvericin was determined in selected extracts; high-resolution product ion spectra were acquired using ESI-UHR-Q-TOF (Bruker Impact II Ultra-High Resolution-TOF) to confirm the presence of Beauvericin. The system ionization source type was ESI with positive ion polarity. The instrument was run in the full-scan mode (*m*/*z* 150–1200). Nitrogen was used for drying (200 °C; 7 L/min), and the nebulizer gas pressure was 1.7 Bar. Other typical operating parameters were set as capillary voltage—4500 V, end plate offset—500 V, and charging voltage—2000 V.

### 2.8. Large Scale Fermentation and Extraction of Beauvericin from Fusarium tardicrescens NFCCI 5201

Among the 88 tested isolates, the highest beauvericin producer, *F. tardicrescens* NFCCI 5201, was subjected to flask scale fermentation in a total of 3.5 L of *Fusarium* defined medium (FDM). Each flask was inoculated with 1% spore suspension of O.D. 0.08–0.1, as described earlier in Section 2.5, and incubated at 25 °C with 150 rpm for one week. Extraction of beauvericin was carried out as described in Section 2.6. The final dry residue was dissolved in 36 mL of acetonitrile. This extract was used for further study.

### 2.9. Determination of the Antibacterial Activity

The *Fusarium tardicrescens* NFCCI 5201 extract containing BEA dissolved in acetonitrile (500 µg/mL) was used to check antimicrobial activity. The antibacterial activity was tested against a panel of test organisms, including *Escherichia coli* MTCC 739, *Bacillus subtilis* MTCC 121, *Micrococcus luteus* MTCC 2470, *Raoultella planticola* MTCC 530, *Pseudomonas aeruginosa* MTCC 2453, *Staphylococcus aureus* MLS16 MTCC 2940 and *Staphylococcus aureus* MTCC 96. Cultures were grown on nutrient agar plates and incubated at 37 °C for 48 h. After incubation, one to two pure colonies of each culture were transferred to 10 mL Muller hinton broth (MHB) and incubated for 4–5 h at 37 °C. Further, optical density was adjusted to 1 using broth. The cultures were diluted to a cell density of 1 × 10^6^ cells/mL. The test was carried out using a resazurin-based turbidometric assay in a 96-well microtiter plate. All wells of rows (A–G) were filled with 100 µL Muller hinton broth. The first well of each row was filled with 100 µL of *Fusarium tardicrescens* NFCCI 5201 extract containing BEA (500 µg/mL), which was mixed well, and then 100 µL of the mixture from the first well was transferred to the second well of the vertical row. This serial dilution was continued until the tenth well; lastly, 100 µL of the mixture was discarded from the tenth well. The final concentration of the extract was half of the original concentration in each well. Ten µL of each bacterial suspension was added in vertical rows individually, except for the twelfth well (sterility control). One hundred µL of antibiotic ampicillin (10 µg/50 µL) was used, except *Pseudomonas aeruginosa* MTCC 2453, in which neomycin (30 µg/50 µL) was added in the eleventh well of each row (positive control). After 48 hrs of incubation at 37 °C, 25 µL of resazurin (0.01%) was added to all wells and incubated at 37 °C for 75 min in the dark. Color changes were observed and recorded. The lowest concentration before the color change was considered as the minimum inhibitory concentration for that particular test organism [47,48].

### 2.10. Determination of the Individual and Combined Effect of Fusarium tardicrescens NFCCI 5201 Extract Containing Beauvericin and Amphotericin B on Pathogenic Fungi of Agricultural Importance

The antifungal potential of the individual and combined effect of *F. tardicrescens* NFCCI 5201 extract containing beauvericin and amphotericin B on agriculturally important pathogenic fungi was carried out using the poisoned food technique as described earlier [49]. Fungal pathogens *Pythium* sp. NFCCI 3482, *Geotrichum candidum* NFCCI 3744, *Rhizoctonia solani* NFCCI 4327, *Rhizopus* sp. NFCCI 2108, *Sclerotium rolfsii* NFCCI 4263, *Bipolaris sorokiniana* NFCCI 4690 and *Absidia* sp. NFCCI 2716 were selected for the antifungal study. These pathogenic fungi were grown on PDA and incubated at 25 ± 2 °C for 5–7 days.

Agar discs with mycelia (5 mm in diameter) were cut out from the actively growing regions of the 5–7 days old pure cultures using a sterile cork borer and aseptically inoculated at the center of four different Petri plates, as follows:

1. Control plate containing PDA;
2. PDA supplemented with amphotericin B (40 µg/mL);
3. PDA supplemented with *F. tardicrescens* NFCCI 5201 extract containing beauvericin (40 µg/mL);
4. PDA supplemented with amphotericin B (20 µg/mL) and *F. tardicrescens* NFCCI 5201 extract containing beauvericin (20 µg/mL).

The growth of fungal colonies on different media was measured between 3 and 7 days depending upon the growth of fungi. Each treatment was repeated thrice, and the plates were incubated at 25 °C. The diameter of the fungal colony was measured after 3 and 7 days of incubation. The percentage inhibition of the mycelial growth of the test fungi was calculated using the formula described earlier [50]. Inhibition of mycelial growth (%) = (*dc*−*dt*)/*dc* × 100, where *dc* is the mean diameter (in mm) of the colony in the control sample and dt is the diameter (in mm) of the colony in the treatment.

### 2.11. Antioxidant Activity of Extract of Fusarium tardicrescens NFCCI 5201 Containing Beauvericin

The extract was tested for in vitro antioxidant activity using the 2,2-diphenyl-1-picrylhydrazyl (DPPH) radical scavenging method.

The concentration of the extract as well as standard solutions (control) glucose (Himedia) (negative) and ascorbic acid (Sigma) (positive) used were 1, 0.9, 0.8, 0.7, 0.6, 0.5, 0.4, 0.3, 0.2 and 0.1 mg/mL in acetonitrile. The extract or standard solution (10 µL) was added to DPPH in acetonitrile solution (200 µL, 100 µM) in a 96-well microtiter plate (Tarsons Products (P) Ltd., Kolkata, India). After incubation at 37 °C for 30 min, the absorbance of each solution was determined at 517 nm using a Synergy HT Multi-detection microplate reader (BioTek, Winooski, VT, USA).

The radical scavenging activity was calculated by the following formula:

Radical scavenging activity (%) = (OD control − OD sample)/OD control × 100 [51,52]. Three replicates were maintained for each treatment.

## 3. Results

### 3.1. Phylogenetic Analysis

**Figure 3:**
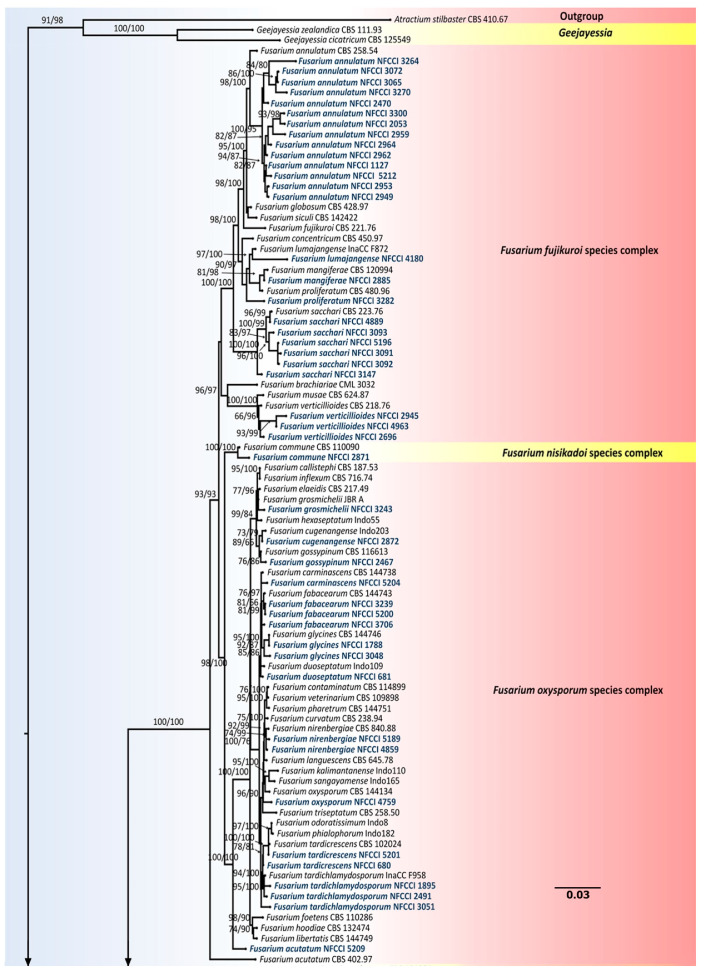

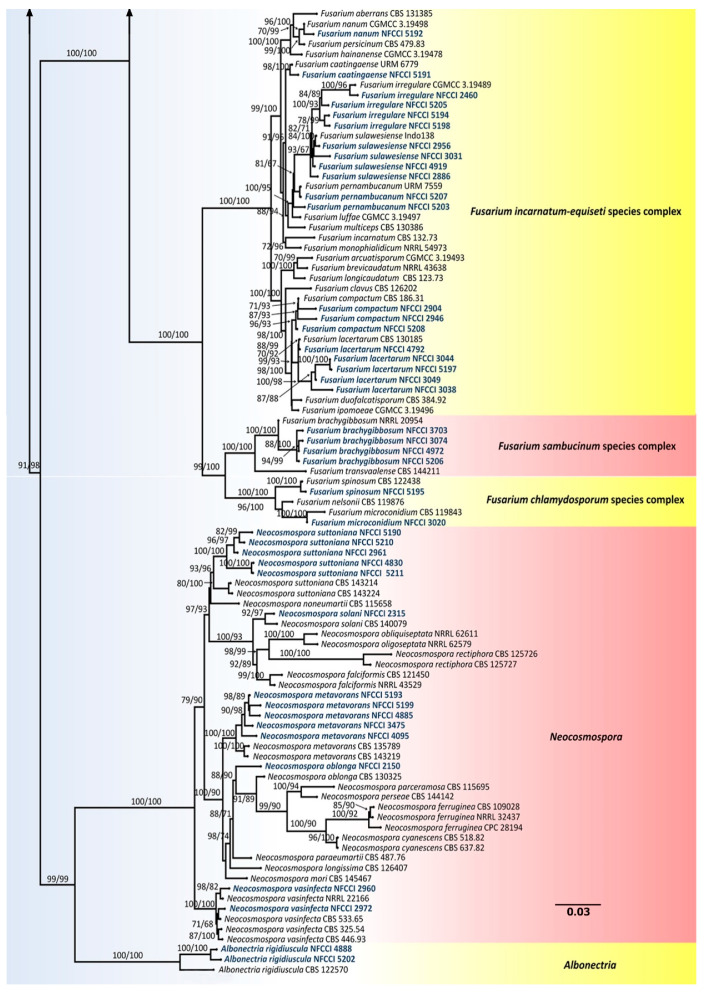
Maximum-likelihood (IQ-TREE-ML) consensus tree inferred from the combined ITS, LSU, *Rpb2*, *β-tub*, *CaM* and *Tef-1α* multiple sequence alignment of genus *Fusarium* and related genera. Numbers at the branches indicate statistical support values (UFBS and SH-aLRT). The scale bar indicates expected changes per site. The tree is rooted to *Atractium stilbaster* (CBS 410.67). Isolates used in this study are shown in blue bold.

The nrDNA (ITS), translation elongation factor 1-alpha (*Tef1-α*), 28S large subunit of the nrDNA (LSU), RNA polymerase second largest subunit (*Rpb2*), beta-tubulin (*β-tub*) and calmodulin (*CaM*) loci sequence alignments were together used to confirm the resolution of the isolates used in this study. The concatenated file contained sequence data of 186 taxa. Alignment contained 6944 columns, 3815 distinct patterns, 2049 parsimony-informative, 950 singleton sites and 3944 constant sites. TIM2e + R4 was found to be the best-fit model of 286 models tested and was chosen based on the Bayesian Information Criterion (BIC). The phylogeny was inferred using the Maximum Likelihood Method based on the model mentioned above. The log-likelihood of the consensus tree was –59368.419. Rate parameters: A-C: 1.27988, A-G: 2.69656, A-T: 1.27988, C-G: 1.00000, C-T: 5.18110, G-T: 1.00000; Base frequencies: A: 0.250, C: 0.250, G: 0.250, T: 0.250; Site proportion and rates: (0.587, 0.115) (0.171, 1.101) (0.200, 2.369) (0.042, 6.470) (Figure 3).

Of the isolates, 88 were found to belong to *Fusarium* and related genera, i.e., *Albonectria* and *Neocosmospora.* Strains used in this study were found to represent 35 species, including *Albonectria rigidiuscula*, *Fusarium acutatum*, *F. annulatum*, *F. brachygibbosum*, *F. caatingaense*, *F. carminascens*, *F. commune*, *F. compactum*, *F. cugenangense*, *F. duoseptatum*, *F. fabacearum*, *F. glycines*, *F. gossypinum*, *F. grosmichelii*, *F. irregulare*, *F. lacertarum*, *F. lumajangense*, *F. mangiferae*, *F. microconidium*, *F. nanum*, *F. nirenbergiae*, *F. oxysporum*, *F. pernambucanum*, *F. proliferatum*, *F. sacchari*, *F. spinosum*, *F. sulawesiense*, *F. tardichlamydosporum*, *F. tardicrescens*, *F. verticillioides*, *Neocosmospora metavorans*, *N. oblonga*, *N. solani*, *N. suttoniana* and *N. vasinfecta*.

*Fusarium* isolates were found to represent six species complexes. The species falling into a particular species complex are included in brackets, viz. *Fusarium sambucinum* species complex [*F. brachygibbosum*], *Fusarium chlamydosporum* species complex [*F. microconidium*, *F. spinosum*], *Fusarium incarnatum-equiseti* species complex [*F. caatingaense*, *F. compactum*, *F. irregulare*, *F. lacertarum*, *F. nanum*, *F. pernambucanum*, *F. sulawesiense*], *Fusarium oxysporum* species complex [*F. carminascens*, *F. cugenangense*, *F. duoseptatum*, *F. fabacearum*, *F. glycines*, *F. gossypinum*, *F. grosmichelii*, *F. nirenbergiae*, *F. oxysporum*, *F. tardichlamydosporum*, *F. tardicrescens*], *Fusarium nisikadoi* species complex [*F. commune*] and *Fusarium fujikuroi* species complex [*F. acutatum*, *F. annulatum*, *F. lumajangense*, *F. mangiferae*, *F. proliferatum*, *F. sacchari*, *F. verticillioides*].

Interestingly, out of 35 species reported in this study, 17 species, *Fusarium caatingaense*, *F. carminascens*, *F. compactum*, *F. cugenangense*, *F. duoseptatum*, *F. fabacearum*, *F. glycines*, *F. gossypinum*, *F. grosmichelii*, *F. lumajangense*, *F. microconidium*, *F. nanum*, *F. nirenbergiae*, *F. sulawesiense*, *F. tardichlamydosporum*, *F. tardicrescens*, *Neocosmospora oblonga* and *N. suttoniana*, were found to be new records from India [53,54], most of them being reported for the first time from a new host in this study (Table 4).

### 3.2. Detection of Beauvericin Produced

#### 3.2.1. Thin Layer Chromatography

**Figure 4:**
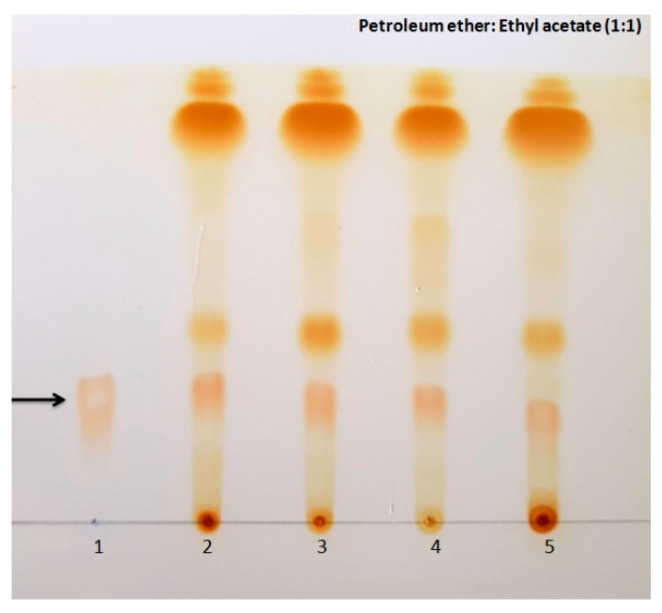
Thin layer chromatography developed in petroleum ether: Ethyl acetate (1:1) showing spots of beauvericin detected using iodine vapours. Black arrow shows spots of beauvericin (1) Standard beauvericin (500 µg/mL) (2) *Fusarium tardicrescens* NFCCI 5201 (3) *F. cugenangense* NFCCI 2872 (4) *Neocosmospora vasinfecta* NFCCI 2960 (5) *Fusarium annulatum* NFCCI 2962.

Thin layer chromatography analysis of fungal extracts along with the standard beauvericin showed spots at the same retention factor as the standard beauvericin, depicting the possible presence of beauvericin in the fungal extracts (Figure 4).

#### 3.2.2. High-Performance Liquid Chromatography

**Figure 5:**
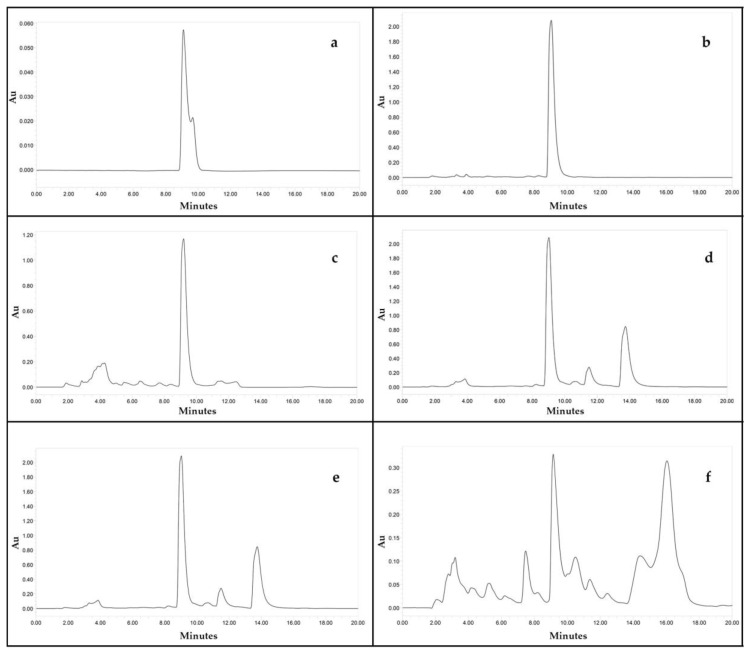
HPLC chromatograms of (**a**) standard beauvericin (500 µg/mL) (**b**) *Fusarium fabacearum* NFCCI 5200 extract (**c**) *F. sacchari* NFCCI 4889 extract (**d**) *F. carminascens* NFCCI 5204 extract (**e**) *F. tardicrescens* NFCCI 5201 extract (**f**) *F. cugenangense* NFCCI 2872 extract.

**Figure 6:**
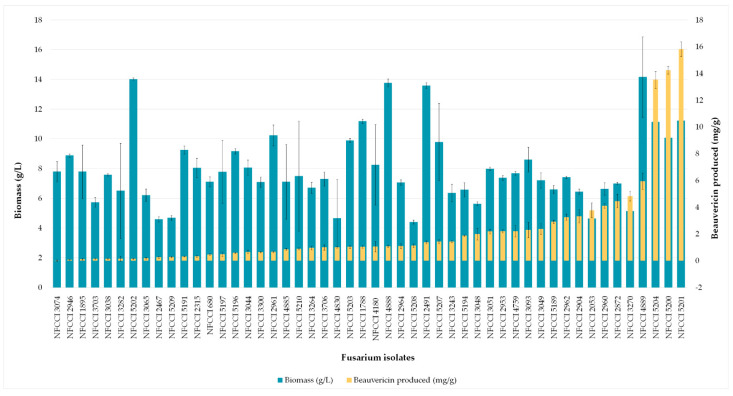
Mycelial biomass and BEA content produced by different *Fusarium* isolates in the *Fusarium* defined medium (on day 7).

There was a linear correlation between the concentration of the standard beauvericin and the areas of the peak in HPLC chromatogram. The retention time of standard beauvericin was found to be 9.1 min (Figure 5). Biomass produced by various isolates varied from 4.41 to 14.17 g/L of FDM. Among 88, 50 isolates (56%) were found to be capable of beauvericin production which varied from 0.01 to 15.82 mg/g of biomass. Table 5 shows the mycelial biomass, BEA content of mycelial biomass and final medium pH after a week’s fermentation of different *Fusarium* isolates in *Fusarium* defined medium. *F. tardicrescens* NFCCI 5201 showed maximum beauvericin production of 15.82 mg/g of biomass. *F. carminascens* NFCCI 5204 and *F. fabacearum* NFCCI 5200 also produced a significant amount of beauvericin: 13.54 and 14.25 mg/g of biomass, respectively (Figure 6). The final pH of the fermented broth varied from 6.38 to 8.96 for different isolates. The correlation coefficient between biomass produced and the beauvericin produced was 0.2692; that between the pH of the fermented broth and the beauvericin produced was 0.16, indicating very weak and/or no association in both cases.

#### 3.2.3. High-Resolution Mass Spectrometry (HRMS)

**Figure 7:**
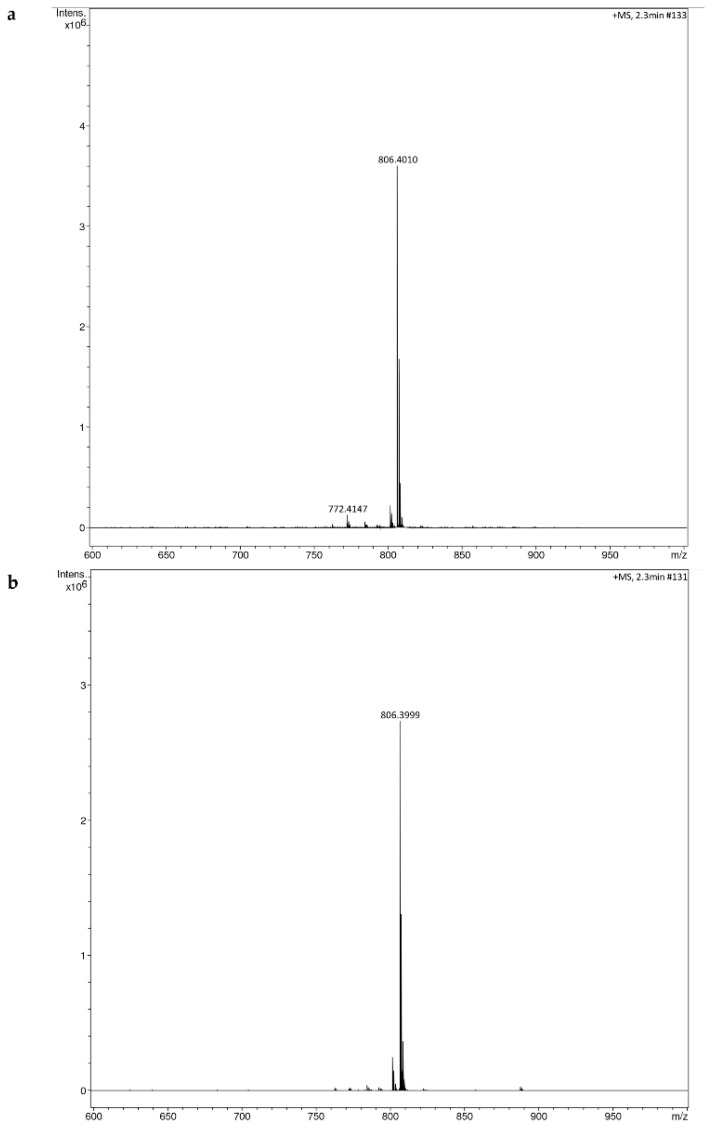
HRMS spectra from higher-collision dissociation of the [M + Na]^+^ ions of fungal extract, (**a**) *Fusarium sacchari* NFCCI 4889 (**b**) *F. tardicrescens* NFCCI 5201.

The HRMS results showed molecular ion peaks at 806.3999 (*m*/*z*) [M + Na]^+^,indicating the presence of beauvericin (C_45_H_57_N_3_O_9_) in the fungal extracts (Figure 7). Similar (*m*/*z*) [M + Na]^+^ 806.3956 have been reported for beauvericin (C_45_H_57_N_3_O_9_) [71]. It was observed that the fungal extract contains, majorly, beauvericin, as maximum ion intensity was observed with *m*/*z* 806.3999.

### 3.3. Large Scale Fermentation

Fermentation of 3.5 liters of *Fusarium* defined medium resulted in 36.27 gms of biomass which, on extraction, resulted in 144 mg of crude which was dissolved in 36 mL of acetonitrile, making the final concentration 4 mg/mL. This extract was subjected to HPLC; it was found that beauvericin is the major compound in the extract, as the area covered by it was found to be nearly 64–68%. This stock was diluted as per requirement for further studies.

### 3.4. Antimicrobial and MIC of Crude Extract of Fusarium tardicrescens NFCCI 5201

**Figure 8:**
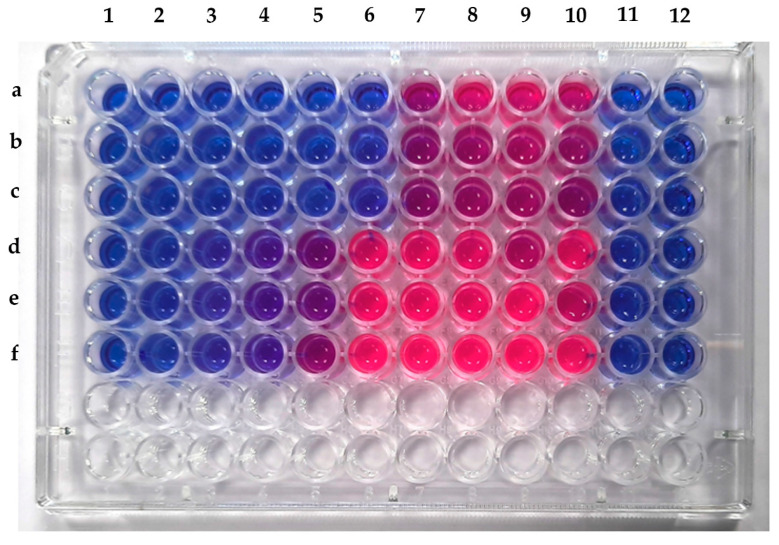
Microtiter plate showing MIC of crude extract of *F*. *tardicrescens* NFCCI 5201 against *Staphylococcus aureus* MLS16 MTCC 2940 (Rows a–c) and *Micrococcus luteus* MTCC 2470 (Rows d–f).

The extract showed promising results against *Staphylococcus aureus* MLS16 MTCC 2940 and *Micrococcus luteus* MTCC 2470. The MIC of the crude extract of *F*. *tardicrescens* NFCCI 5201 was found to be 15.63 µg/mL against *Micrococcus luteus* MTCC 2470 and 62.5 µg/mL against *Staphylococcus aureus* MLS16 MTCC 2940 (Figure 8). The extract did not show any antimicrobial activity against *Escherichia coli* MTCC 739, *Bacillus subtilis* MTCC 121, *Raoultella planticola* MTCC 530, *Pseudomonas aeruginosa* MTCC 2453 and *Staphylococcus aureus* MTCC 96.

### 3.5. Individual and Combined Effect of Extract of Fusarium tardicrescens NFCCI 5201 Containing Beauvericin and Amphotericin B on Pathogenic Fungi

**Figure 9:**
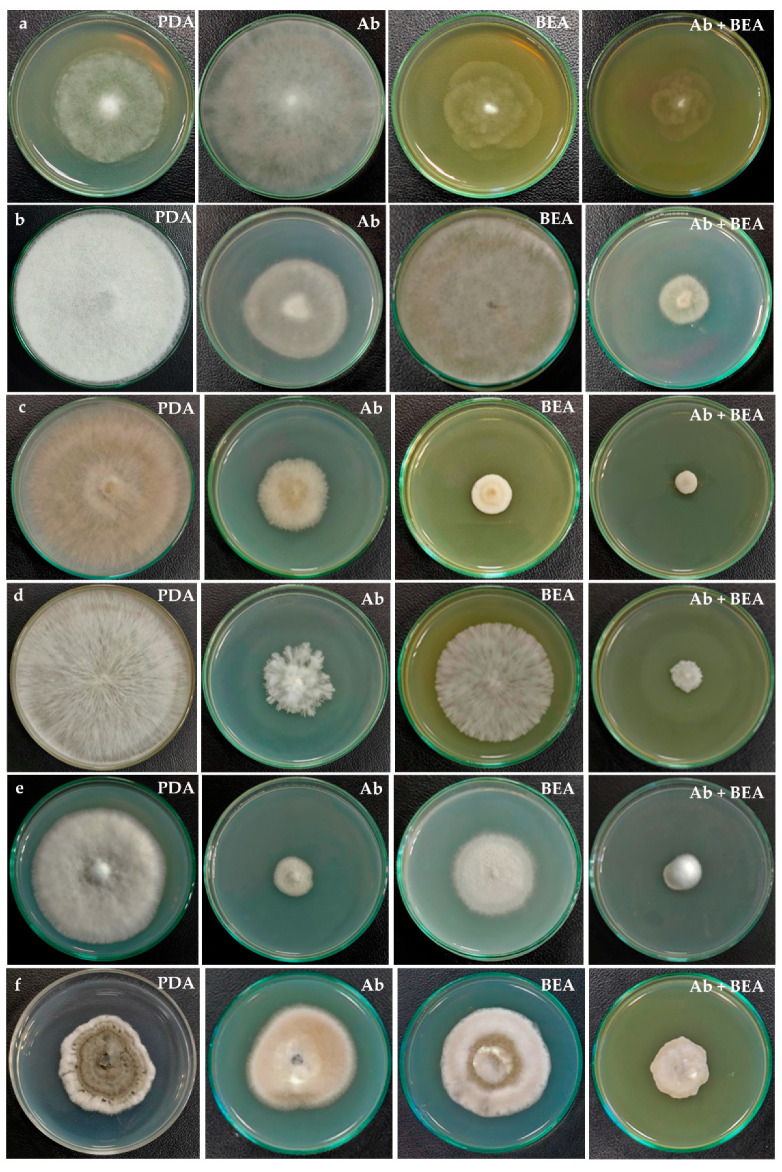
Antifungal activity of individual and combined effect of extract of *F*. *tardicrescens* NFCCI 5201 containing beauvericin and amphotericin B on various pathogenic fungi (**a**) *Pythium* sp. NFCCI 3482 (**b**) *Rhizopus* sp. NFCCI 2108 (**c**) *Rhizoctonia solani* NFCCI 4327 (**d**) *Sclerotium rolfsii* NFCCI 4263 (**e**) *Geotrichum candidum* NFCCI 3744 (**f**) *Bipolaris sorokiniana* NFCCI 4690. PDA: Potato dextrose agar (Control); Ab: Potato dextrose agar containing amphotericin B (40 µg/mL); BEA: Potato dextrose agar having extract of *F*. *tardicrescens* NFCCI 5201 containing beauvericin (40 µg/mL); Ab + BEA: Potato dextrose agar having amphotericin B (20 µg/mL) and extract of *F*. *tardicrescens* NFCCI 5201 containing beauvericin (20 µg/mL).

**Figure 10:**
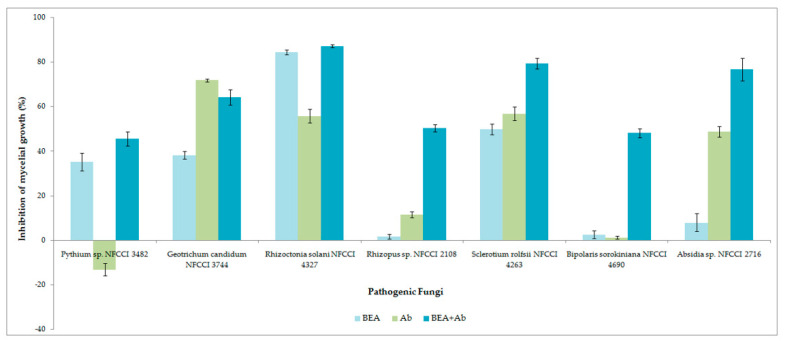
Inhibition of mycelial growth (%) of individual and combined effect of extract of *F. tardicrescens* NFCCI 5201 containing beauvericin and amphotericin B on various pathogenic fungi using food poison technique.

The antifungal activity was observed as a reduction in the mycelial growth of pathogenic fungi in poisoned plates when compared to the control plates (Figure 9). The extract of *F.* *tardicrescens* NFCCI 5201 containing beauvericin (40 µg/mL) showed good antifungal activity against plant pathogenic fungi, *Rhizoctonia solani* NFCCI 4327, *Sclerotium rolfsii* NFCCI 4263, *Geotrichum candidum* NFCCI 3744 and *Pythium* sp. NFCCI 3482 showed a % inhibition of 84.31, 49.76, 38.22 and 35.13. Amphotericin B (40 µg/mL) inhibited *Geotrichum candidum* NFCCI 3744, *Rhizoctonia solani* NFCCI 4327, *Sclerotium rolfsii* NFCCI 4263 and *Absidia* sp. NFCCI 2716, showing % inhibition of 71.76, 55.72, 56.82 and 48.68. Interestingly, the results were remarkable when the extracts of *F.* *tardicrescens* NFCCI 5201 containing beauvericin (20 µg/mL) and amphotericin B (20 µg/mL) were used in combination against *Rhizopus* sp. NFCCI 2108, *Sclerotium rolfsii* NFCCI 4263, *Bipolaris sorokiniana* NFCCI 4690 and *Absidia* sp. NFCCI 2716, showing % inhibition of 50.35, 79.37, 48.07 and 76.72%, respectively. Individually, extract of *F*. *tardicrescens* NFCCI 5201 containing beauvericin (40 µg/mL) showed % inhibition of 1.68, 49.76, 2.56 and 7.9 against *Rhizopus* sp. NFCCI 2108, *Sclerotium rolfsii* NFCCI 4263, *Bipolaris sorokiniana* NFCCI 4690 and *Absidia* sp. NFCCI 2716. Individually, amphotericin B (40 µg/mL) showed % inhibition of 11.51, 56.82, 1.28 and 48.67 against *Rhizopus* sp. NFCCI 2108, *Sclerotium rolfsii* NFCCI 4263, *Bipolaris sorokiniana* NFCCI 4690 and *Absidia* sp. NFCCI 2716. The extract of *F.* *tardicrescens* NFCCI 5201 containing beauvericin (40 µg/mL) showed better results than amphotericin B in the case of *Pythium* sp. NFCCI 3482 and *Rhizoctonia solani* NFCCI 4327 (Figure 10).

### 3.6. Antioxidant Effect of Extract of Fusarium tardicrescens NFCCI 5201 Containing Beauvericin

**Figure 11:**
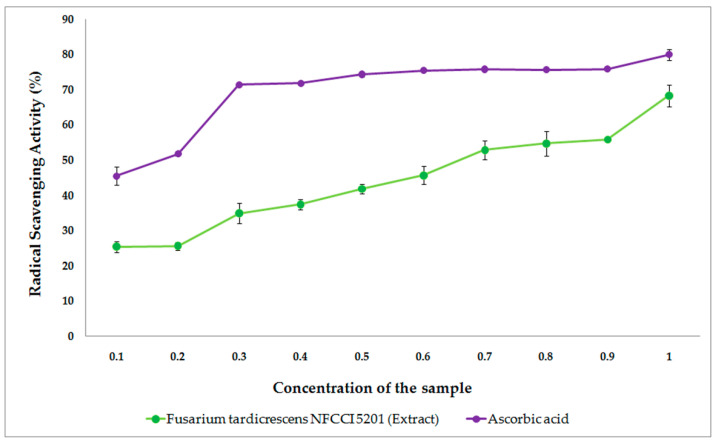
The free radical scavenging activity of the extract of *F*. *tardicrescens* NFCCI 5201 containing beauvericin (mg/mL) and the standard ascorbic acid (mg/mL) when tested for in vitro antioxidant activity.

Figure 11 shows the free radical scavenging activity of the extract of *F. tardicrescens* NFCCI 5201 containing beauvericin and the standard ascorbic acid when tested for in vitro antioxidant activity. The extract of *F*. *tardicrescens* NFCCI 5201 containing beauvericin showed good satisfactory dose-dependent DPPH radical scavenging activity with an IC_50_ value of 0.675 mg/mL when tested with standard ascorbic acid, which showed IC_50_ value of 0.146 mg/mL.

## 4. Discussion

One of the greatest hindrances in studying *Fusarium* has been the ambiguous nomenclature or incorrect species names of the isolates because of the limitations in recognition of the species based on morphology [72]. Being the world’s most important pathogens, its prophylaxis and management needs correct and rapid identification [73]. As mentioned earlier, ITS and LSU frequently fail to distinguish at the species level and, preferably, *Tef1-α* and *Rpb2* can be used to distinguish isolates at the species level. Literature reveals that Indian *Fusarium* isolates have been mostly identified on the basis of morphology. Very few studies were found where the *Fusarium* isolates had been identified using molecular studies. Wherever molecular studies were performed, they were based on the ITS gene region; very limited studies used either *Tef1-α* or *Rpb2* or both of these gene regions [74]. To our knowledge, this is the first study from India where *Fusarium* isolates have been identified on the basis of six gene regions, nrDNA (ITS), *translation elongation factor 1-alpha* (*Tef1-α*), 28S large subunit of the nrDNA (LSU), *RNA polymerase second largest subunit* (*Rpb2*), *beta-tubulin* (*β-tub*) and *calmodulin* (*CaM*). Interestingly, in this study, 17 species, *Fusarium caatingaense*, *F. carminascens*, *F. compactum*, *F. cugenangense*, *F. duoseptatum*, *F. fabacearum*, *F. glycines*, *F. gossypinum*, *F. grosmichelii*, *F. lumajangense*, *F. microconidium*, *F. nanum*, *F. nirenbergiae*, *F. sulawesiense*, *F. tardichlamydosporum*, *F. tardicrescens*, *Neocosmospora oblonga* and *N. suttoniana* were found to be new records for India, most of them being reported from new hosts [53,54].

BEA is a potent bioactive compound possessing antimicrobial, anti-insecticidal, antitumor and antiplatelet activities at extremely low concentration and with unique uncharacterized active mechanisms. A review of the literature indicates that no study was undertaken in India on BEA production from Indian species of *Fusarium,* except [43] who had reported BEA production from two isolates of *Fusarium* viz., *F*. *anthophilum* (Host: Sugarcane; 1300 µg/g) and *F*. *nygamai* (Host: *Cajanus indicus*; 3 µg/g) [43]. This work was undertaken in Bari, Italy. This suggests that Indian *Fusarium* isolates have remained unexplored for their capability of BEA production and its applicability.

*Albonectria rigidiuscula*, *F. acutatum*, *F. annulatum*, *F. brachygibbosum*, *F. caatingaense*, *F. carminascens*, *F. compactum*, *F. cugenangense*, *F. fabacearum*, *F. glycines*, *F. gossypinum*, *F. grosmichelii*, *F. irregulare*, *F. lacertarum*, *F. lumajangense*, *F. nirenbergiae*, *F. oxysporum*, *F. pernambucanum*, *F. proliferatum*, *F. sacchari*, *F. tardichlamydosporum*, *F. tardicrescens*, *N. metavorans*, *N. solani*, *N. suttoniana* and *N. vasinfecta* were found to be the positive producers of BEA in this study, many of them being reported as BEA producers for the first time in this study. *Fusarium tardicrescens* NFCCI 5201 showed maximum beauvericin production of 15.82 mg/g of biomass. *Fusarium carminascens* NFCCI 5204 and *F. fabacearum* NFCCI 5200 also produced a significantly ample amount of beauvericin, 13.54 and 14.25 mg/g of biomass, respectively, all three of them being reported for the first time as significant BEA producers in this study.

In earlier reports, BEA was detected in cultures of *Fusarium moniliforme*, *F. semitectum* [75], *F. subglutinans, F. thapsinum* [76], *F. sambucinum*, *F. acuminatum*,* F. equiseti*,* F. longipes*,* F. anthophilum*,* F. oxysporum*,* F. poae*,* F. avenaceum*,* F. beomiforme*,* F. dlamini*,* F. bulbicola*,* F. nygamai* [43], *F. chlamydosporum*,* F. solani*,* F. proliferatum* and *F. sacchari* [77].

The crude extract of *F*. *tardicrescens* NFCCI 5201 showed promising results against *Staphylococcus aureus* MLS16 MTCC 2940 and *Micrococcus luteus* MTCC 2470, with MIC values of 15.63 µg/mL against *Micrococcus luteus* MTCC 2470 and 62.5 µg/mL against *Staphylococcus aureus* MLS16 MTCC 2940. BEA from *F. oxysporum* had a potent inhibitory effect on the growth of pathogenic *Staphylococcus aureus* with a MIC of 3.91 µM [23]. Earlier reports reveal that inhibitory concentration (IC_50_) values of BEA against *Bacillus subtilis*, *Staphylococcus haemolyticus*, *Pseudomonas lachrymans*, *Agrobacterium tumefaciens*, *Escherichia coli* and *Xanthomo vesicatoria* by a 96-well microplate broth dilution–MTT assay ranged between 18.45 and 70.41 µg/mL [78]. BEA has been known to inhibit bacteria, including *Bacillus pumilus* (0.1 µg of BEA per disk), several other species of *Bacillus* and *Paenibacillus* (1 µg of BEA per disk), *P*. *validus*, *Bifidobacterium adolescentis*, *Clostridium perfringens*, *Eubacterium biforme*, *Peptostreptococcus anaerobius* and *P*. *productus* (25 µg of BEA per disk) [18]. Strong antimicrobial activity has been reported against *Staphylococcus aureus*, *Salmonella typhimurium* and *Bacillus cereus* with MIC values of 3.91 µM, 6.25 and 3.12 µg/mL, respectively [19,23].

With BEA being a high score ABC inhibitor, it has been reported to show strong synergy with some azole compounds (miconazole, ketoconazole) against *Candida albicans* and *Candida parapsilosis* (in vitro as well as in vivo). It has been reported that BEA individually fails to show antifungal activity, but it was found to synergize the effect of ketoconazole [25].

In this study, the antifungal activity was observed as a reduction in the mycelial growth of pathogenic fungi of agricultural importance in poisoned plates when compared to the control plates. The extract of *F. tardicrescens* NFCCI 5201 containing beauvericin (40 µg/mL) showed good antifungal activity against pathogenic fungi *Rhizoctonia solani* NFCCI 4327, *Sclerotium rolfsii* NFCCI 4263, *Geotrichum candidum* NFCCI 3744 and *Pythium* sp. NFCCI 3482 showing % inhibition of 84.31, 49.76, 38.22 and 35.13. Interestingly, when the extract of *F. tardicrescens* NFCCI 5201 containing beauvericin (20 µg/mL) and amphotericin B (20 µg/mL) were used in combination, they produced a synergized antifungal effect against *Rhizopus* sp. NFCCI 2108, *Sclerotium rolfsii* NFCCI 4263, *Bipolaris sorokiniana* NFCCI 4690 and *Absidia* sp. NFCCI 2716, showing % inhibition of 50.35, 79.37, 48.07 and 76.72%, respectively. Individually, the extract of *F. tardicrescens* NFCCI 5201 containing beauvericin (40 µg/mL) showed a % inhibition of 1.68, 49.76, 2.56 and 7.9 against *Rhizopus* sp. NFCCI 2108, *Sclerotium rolfsii* NFCCI 4263, *Bipolaris sorokiniana* NFCCI 4690 and *Absidia* sp. NFCCI 2716, and amphotericin B (40 µg/mL) showed a % inhibition of 11.51, 56.82, 1.28 and 48.67 against *Rhizopus* sp. NFCCI 2108, *Sclerotium rolfsii* NFCCI 4263, *Bipolaris sorokiniana* NFCCI 4690 and *Absidia* sp. NFCCI 2716. The extract of *F. tardicrescens* NFCCI 5201 containing beauvericin (40 µg/mL) showed better results than amphotericin B in the case of *Pythium* sp. NFCCI 3482, *Rhizoctonia solani* NFCCI 4327. This is the first report on the testing of BEA against filamentous, agriculturally important fungal pathogens. The synergistic effect of BEA along with amphotericin B against filamentous agriculturally important pathogens has also been studied for the first time. Earlier studies focused on the synergistic effect of BEA and azoles (miconazole, ketoconazole) on yeast (*Candida albicans* and *Candida parapsilosis*). This study focuses on the synergistic effect of fungal extracts and polyene (amphotericin B) on agriculturally important filamentous fungal pathogens. Moreover, the extract of *F. tardicrescens* NFCCI 5201 containing beauvericin showed good dose-dependent DPPH radical scavenging activity with an IC_50_ value of 0.675 mg/mL. Antioxidant activity has been reported from metabolites produced by *Fusarium oxysporum* [79]. The DPPH-radicals’ scavenging activity has been reported from *F. solani* with IC_50_ value of 24 µg/mL [80].

## 5. Conclusions

*Fusarium* spp. have been known globally for centuries for their pathogenic behavior; however, they can prove to be a treasure trove because of their capability to produce other interesting metabolites of great applicability in various sectors such as food, agriculture and medicine. As shown in this study, the antifungal activity became synergized when a half dosage of the fungal extract and amphotericin B was used. Therefore, this study indicates that concerted efforts are required, in which high-throughput screening needs to be carried out with dosage of the BEA and the other antifungal compound, which can possibly be used at a large scale for calculating the optimum dosage required. Similar studies can be carried out in the field of medicine where the dosage is optimized and the delivery of the drug combinations is also targeted.

## Figures and Tables

**Table 1:** Sixty-four *Fusarium* isolates procured from the National Fungal Culture Collection of India (NFCCI), along with other details such as host, place of collection and date of isolation, are listed.

NFCCI No.	Host	Place of Collection	Date of Isolation
NFCCI 680	*Azadirachta indica* (Endophyte)	Maharashtra	25 September 2006
NFCCI 681	*Azadirachta indica* (Endophyte)	Maharashtra	20 December 2006
NFCCI 1127	*Jatropha curcus* (Phyloplane)	Durg, Chhattisgarh	23 May 2007
NFCCI 1788	Soil	Imphal, Manipur	23 June 2009
NFCCI 1895	Soil	Mahabaleshwar, Maharashtra	25 August 2009
NFCCI 2053	Wilted Guava plant (Root)	Rajasthan	9 February 2010
NFCCI 2150	Soil	Arctic Tundra	25 June 2010
NFCCI 2315	Potato	Goa	15 June 2009
NFCCI 2460	*Zanthooxylum armatum*	Udhampur, Jammu and Kashmir	13 June 2011
NFCCI 2467	Bottle Gourd	Dapoli, Maharashtra	16 June 2011
NFCCI 2470	Coconut leaves	Vellayani, Thiruvananthapuram, Kerala	22 June 2011
NFCCI 2491	Soil	Bangalore, Karnataka	8 July 2012
NFCCI 2696	*Coleus* sp.	Puthenthope, Kerala	19 March 2012
NFCCI 2871	Wilted tomato plant (Root)	Manipur	25 September 2012
NFCCI 2872	Wilted tomato plant (Root)	Manipur	25 September 2012
NFCCI 2885	*Azadirachta indica* (Endophyte)	Chandravati, Rajasthan	9 April 2012
NFCCI 2886	*Azadirachta indica* (Endophyte)	Chikkamagaluru, Karnataka	15 July 2012
NFCCI 2904	*Aegle marmelos*	Karnataka	28 November 2009
NFCCI 2945	Soil	Tiruchirappalli, Tamil Nadu	7 January 2012
NFCCI 2946	*Aegle marmelos*	Karnataka	25 November 2010
NFCCI 2949	Cotton field	Shirpur, Maharashtra	9 October 2012
NFCCI 2953	Cotton field	Shirpur, Maharashtra	6 August 2015
NFCCI 2956	Cotton field	Shirpur, Maharashtra	19 October 2012
NFCCI 2959	Pigeon pea (Root)	Amravati, Maharashtra	30 October 2012
NFCCI 2960	Pigeon pea (Root)	Amravati, Maharashtra	30 October 2012
NFCCI 2961	Pigeon pea (Root)	Beed, Maharashtra	26 October 2012
NFCCI 2962	Pigeon pea (Root)	Kalamb, Osmanabad, Maharashtra	26 October 2011
NFCCI 2964	Pigeon pea (Root)	Latur, Maharashtra	30 August 2012
NFCCI 2972	Pigeon pea (Root)	Ahmednagar, Maharashtra	30 October 2012
NFCCI 3020	Soil	Karnataka	24 December 2010
NFCCI 3031	Piper betle (Leaf-endophyte)	Tamil Nadu	26 December 2011
NFCCI 3038	Wilted cumin plant	Gujarat	24 February 2013
NFCCI 3044	Wilted cumin plant	Gujarat	24 February 2013
NFCCI 3048	Wilted cumin plant	Gujarat	24 February 2013
NFCCI 3049	Wilted cumin plant	Gujarat	24 February 2013
NFCCI 3051	Wilted cumin plant	Gujarat	24 February 2013
NFCCI 3065	*Ocimum sanctum*	Maharashtra	20 August 2012
NFCCI 3072	*Barleria prionitis* (Stem)	Maharashtra	24 September 2012
NFCCI 3074	*Barleria prionitis*	Maharashtra	20 September 2012
NFCCI 3091	Textile sludge	Tamil Nadu	14 September 2012
NFCCI 3092	Textile sludge	Tamil Nadu	8 September 2012
NFCCI 3093	Textile sludge	Tamil Nadu	15 September 2012
NFCCI 3147	Rotten sugarcane	Dapoli, Maharashtra	5 January 2013
NFCCI 3239	Marigold (Seeds)	Mumbai, Maharashtra	8 February 2013
NFCCI 3243	Cow pea	Thiruvananthapuram, Kerala	20 June 2012
NFCCI 3264	Cow pea	Thiruvananthapuram, Kerala	12 September 2012
NFCCI 3270	Dead wood	Mumbai, Maharashtra	29 March 2013
NFCCI 3282	Soil	Amarkantak, Madhya Pradesh	26 September 2012
NFCCI 3300	*Cathranthus roseus*	Raipur, Chhattisgarh	12 August 2012
NFCCI 3475	*Ocimum sanctum* (Endophyte)	Amaravati, Maharashtra	21 July 2013
NFCCI 3703	Onion	Nanded, Maharashtra	14 September 2014
NFCCI 3706	*Castor* sp. (Root)	Aanand, Gujarat	20 December 2014
NFCCI 4095	Soil	Pune, Maharashtra	2 April 2014
NFCCI 4180	Soil	Chandigarh	19 June 2018
NFCCI 4759	*Anaphalis contorta* (Root-endophyte)	Imphal, Manipur	18 February 2019
NFCCI 4792	Chickpea (Root)	Pratapgarh, Uttar Pradesh	21 January 2018
NFCCI 4830	Cotton (Root)	Madurai, Tamil Nadu	5 September 2018
NFCCI 4859	*Dillenia indica*	Arunachal Pradesh	15 October 2016
NFCCI 4885	Cucumber (Twig and leaf)	Jobner, Jaipur, Rajasthan	3 May 2020
NFCCI 4888	Cucumber (Twig)	Jobner, Jaipur, Rajasthan	02 June 2020
NFCCI 4889	Unknown insect	Vishakhapatnum Andhra Pradesh	30 October 2020
NFCCI 4919	Soil	Pathanamthitta, Kerala	15 September 2020
NFCCI 4963	Plant litter	Raiganj, Uttar dinajpur, West Bengal	25 March 2019
NFCCI 4972	Sugarcane bagasse	Dharur, Beed, Maharashtra	22 December 2020

**Table 2:** Twenty-four isolates isolated in this study along with their accession nos., host, place of collection, date of collection and date of isolation details.

NFCCI No.	Host	Place of Collection	Date of Collection	Date of Isolation
NFCCI 5189	Soil	Haridwar, Uttarakhand	2 July 2018	12 July 2018
NFCCI 5190	Ginger	Pune, Maharashtra	27 August 2017	08 September 2017
NFCCI 5191	Pomegranate	Pune, Maharashtra	7 September 2019	15 September 2019
NFCCI 5192	*Zingiber officinale*	Aurangabad, Maharashtra	9 March 2017	10 March 2017
NFCCI 5193	Soil	Jalgaon, Maharashtra	10 October 2017	14 October 2017
NFCCI 5194	Forest litter	Mahabaleshwar, Maharashtra	12 October 2017	14 October 2017
NFCCI 5195	Soil	Chandigarh	1 April 2019	19 April 2019
NFCCI 5196	Wilted sugarcane plant	Gorakhpur, Uttar Pradesh	2 July 2021	17 July 2021
NFCCI 5197	*Chrysanthemum roseum*	Mahabaleshwar, Maharashtra	25 September 2018	27 September 2018
NFCCI 5198	Soil	Pune, Maharashtra	5 March 2020	16 March 2020
NFCCI 5199	Soil	Jalgaon, Maharashtra	8 October 2017	14 October 2017
NFCCI 5200	Pea	Pune, Maharashtra	15 April 2019	21 April 2019
NFCCI 5201	Dead bark	Simbal, Himachal Pradesh	14 December 2017	25 December 2017
NFCCI 5202	Wilted Cashew plant	Pilcode, Kerala	6 August 2019	20 August 2019
NFCCI 5203	Rotten mushroom	Simbal, Himachal Pradesh	19 May 2019	25 May 2019
NFCCI 5204	Potato	Pune, Maharashtra	28 December 2018	12 January 2019
NFCCI 5205	*Aloe vera* (Leaf)	Pune, Maharashtra	5 October 2019	16 October 2019
NFCCI 5206	Soil	Vadodara, Gujarat	3 October 2019	15 October 2019
NFCCI 5207	Coconut	Goa	15 October 2018	29 October 2018
NFCCI 5208	*Zingiber officinale*	Aurangabad, Maharashtra	5 October 2018	12 March 2018
NFCCI 5209	Carrot rhizosphere	Pithoragarh, Uttarakhand	3 October 2019	7 October 2019
NFCCI 5210	Chilli (Fruit)	Simbal, Himachal Pradesh	5 February 2019	12 February 2019
NFCCI 5211	Damping-off Onion	Udaipur, Rajasthan	19 September 2019	27 September 2019
NFCCI 5212	Grape (Leaf)	Nashik, Maharashtra	16 March 2020	17 April 2020

**Table 3:** PCR primer sets used in this study for amplification as well as sequencing.

Name of the Gene Region	Primer	Direction	Sequence (5′-3′)	Reference
Internal transcribed spacer region of the nrDNA (ITS)	ITS-5	Forward	GGAAGTAAAAGTCGTAACAAGG	[30]
	ITS-4	Reverse	TCCTCCGCTTATTGATATGC	[30]
Translation elongation factor 1-alpha (*tef1-α*)	EF-1	Forward	ATGGGTAAGGARGACAAGAC	[31]
	EF-2	Reverse	GGARGTACCAGTSATCATG	[31]
28S large subunit of the nrDNA (LSU)	LR-OR	Forward	ACCCGCTGAACTTAAGC	[32]
	LR-7	Reverse	TACTACCACCAAGATCT	[33]
RNA polymerase second largest subunit (*rpb2*)	fRPB2-5f	Forward	GAYGAYMGWGATCAYTTYGG	[34]
	fRPB2-7cR	Reverse	CCCATRGCTTGYTTRCCCAT	[34]
Beta-tubulin (*β-tub*)	βtuFFo1	Forward	CAGACCGGTCAGTGCGTAA	[35]
	βtuFRo1	Reverse	TTGGGGTCGAACATCTGCT	[35]
Calmodulin *(CaM*)	CF-1	Forward	GCCGACTCTTTGACYGARGAR	[36]
	CF-4	Reverse	TTTYTGCATCATRAGYTGGAC	[36]

**Table 4:** New records of *Fusarium* from India along with their host detail.

Identity	This Study		Earlier Reports	
	NFCCI No.	Host	Host	Reference
*Fusarium caatingaense*	NFCCI 5191	Pomegranate	*Dactylopius opuntiae*	[55]
*Fusarium carminascens*	NFCCI 5204	Potato	*Zea mays*	[56]
*Fusarium compactum*	NFCCI 2946	*Aegle marmelos*	*Poa annua* Soil Spinach Wild rocket Cultivated rocket Lettuce *Quercus suber* Wheat Apple fruit cultivar Idared and Pink lady Banana corm and root rot Safflower (*Carthamus tinctorius L.*) Wheat soil Grasses	[https://www.ncbi.nlm.nih.gov/nuccore accessed on 29 April 2022, [57,58,59,60,61,62]]
	NFCCI 2904			
	NFCCI 5208	*Zingiber officinale*		
*Fusarium cugenangense*	NFCCI 2872	Wilted tomato plant (Root)	*Crocus* sp. *Gossypium barbadense* Human toe nail *Vicia faba* *Musa* sp. var. Pisang Kepok *Gossypium* sp.	[56,63]
*Fusarium duoseptatum*	NFCCI 681	*Azadirachta indica* (Endophyte)	*Musa sapientum* cv. Pisang Ambon *Musa* sp. var. Pisang Rastali *M. acuminata* var. Dwarf Cavendish *M. acuminata* var. Pisang Ambon *Musa* sp. var. Pisang Raja *Musa* sp. var. Pisang Hawa *Musa* sp. var. Pisang Awak *Musa* sp. var. Pisang Susu *Musa* sp. var. Pisang Keling	[56,63]
*Fusarium fabacearum*	NFCCI 3239	Marigold seeds	*Zea mays* *Glycine max*	[56]
	NFCCI 3706	Castor root		
	NFCCI 5200	Pea		
*Fusarium glycines*	NFCCI 3048	Wilted cumin plant	*Linum usitatissium* *Ocimum basilicum* *Glycine max*	[56]
	NFCCI 1788	Soil		
*Fusarium gossypinum*	NFCCI 2467	Bottle gourd	*Gossypium hirsutum*	[56]
*Fusarium grosmichelii*	NFCCI 3243	Cow pea	Banana corm *M. acuminata* var. Pisang Ambon *Musa* sp. var. Pisang Awak *M. acuminata* var. Pisang Ambon Lumut *M. acuminata* var. Cavendish *Musa* sp. var. Pisang Siem Jumbo *M. acuminata* var. Pisang Ambon Kuning *Musa* sp. var. Pisang Kepok	[63,64]
*Fusarium lumajangense*	NFCCI 4180	Soil	*Musa* sp. var. Pisang Raja Nangka *Musa acuminata* var. Pisang Mas Kirana	[65]
*Fusarium microconidium*	NFCCI 3020	Soil	Unknown	[11]
*Fusarium nanum*	NFCCI 5192	*Zingiber officinale*	*Musa nana* (Leaves) *Solanum lycopersicum* *Musa acuminata* Oat Soil *Triticum* sp. *Sorghum* sp.	[[66], https://www.ncbi.nlm.nih.gov/nuccore accessed on 29 April 2022]
*Fusarium nirenbergiae*	NFCCI 5189	Soil	*Secale cereale* *Musa* sp. *S. tuberosum* *Solanum lycopersicum* *Passiflora edulis* *Chrysanthemum* sp. *Bouvardia longiflora* *Dianthus caryophyllus* *Agathosma betulina* Tulip roots Amputated human toe Human leg ulcer	[56]
	NFCCI 4859	*Dillenia indica*		
*Fusarium sulawesiense*	NFCCI 2956	Cotton field	*Musa acuminata* var. Pisang Cere (AAA) *Oryza* sp. *Smilax corbularia* *Acalypha insulana* *Alocasia odora* *Ipomoea batatas* *Musa nana* *Musa paradisiacal* Plum leaf *Triticum aestivum* Soil *Oryza sativa* *Aleurocanthus woglumi* *Phaseolus lunatus* Eucalyptus *Carica papaya* *Prosopis* sp. *Cucumis melo* *Galia melon* *Bixa orellana* *Gossypium hirsutum* *Sorghum vulgare* *Musa sampientum* var. Robusta Mango leaf Rhizosphere soil (*Bromus tectorum*) Soybean	[[11,67,68,69,70], https://www.ncbi.nlm.nih.gov/nuccore accessed on 29 April 2022]
	NFCCI 2886	*Azadirachta indica* (Endophyte)		
	NFCCI 3031	*Piberbettle leaf* (endophyte)		
	NFCCI 4919	Soil		
*Fusarium tardichlamydosporum*	NFCCI 3051	Wilted cumin plant	*M. sapientum* cv. Pisang Awak Legor *Musa* sp. var. Monthan *M. acuminata* var. Pisang Barangan *Musa* sp. var. Bluggoe *M. acuminata* var. Lady finger *Musa* sp. var. Ney Poovan *Musa* sp. var. Pisang Awak Legor	[56,63]
	NFCCI 1895	Soil		
	NFCCI 2491			
*Fusarium tardicrescens*	NFCCI 680	*Azadirachta indica* (Endophyte)	*Musa* sp. var. Harare *Cicer* sp. *Raphanus* sp.	[63]
	NFCCI 5201	Dead bark		
*Neocosmospora oblonga*	NFCCI 2150	Soil	Human eye Carbonatite	[[11], https://www.ncbi.nlm.nih.gov/nuccore accessed on 29 April 2022]
*Neocosmospora suttoniana*	NFCCI 5190	Ginger	Human wound Equine eye Soil *Homo sapiens* *Gossypium hirsutum* *Podocnemis unifilis* Human cornea Human skin leukemic Human blood leukemia Human blood	[[11], https://www.ncbi.nlm.nih.gov/nuccore accessed on 29 April 2022]
	NFCCI 2961	Pigeon pea root (Wilted)		
	NFCCI 5210	Chilli fruit		
	NFCCI 4830	Cotton rot		
	NFCCI 5211	Onion plants (Damping-off)		

**Table 5:** Mycelial biomass and BEA content of *Fusarium* isolates and their pH in the *Fusarium* defined medium (on day 7).

Identity	NFCCI No.	Biomass (g/L)	BEA Content (mg/g)	Final pH of Medium
*Fusarium nirenbergiae*	NFCCI 5189	6.61 ± 0.26	2.93 ± 0.12	7.73 ± 0.32
*Fusarium annulatum*	NFCCI 3264	6.72 ± 0.37	0.97 ± 0.14	7.48 ± 0.18
*Fusarium lacertarum*	NFCCI 3044	8.07 ± 0.52	0.66 ± 0.14	7.65 ± 0.24
*Neocosmospora suttoniana*	NFCCI 5190	7.14 ± 0.08	-	7.63 ± 0.22
*Fusarium fabacearum*	NFCCI 3239	6.22 ± 0.31	-	7.77 ± 0.4
*Fusarium sacchari*	NFCCI 3147	6.34 ± 0.10	-	7.6 ± 0.06
*Fusarium caatingaense*	NFCCI 5191	9.26 ± 0.26	0.33 ± 0.03	7.63 ± 0.03
*Neocosmospora solani*	NFCCI 2315	8.06 ± 0.65	0.34 ± 0.07	7.61 ± 0.12
*Fusarium annulatum*	NFCCI 3072	6.83 ± 0.09	-	7.75 ± 0.15
*Fusarium glycines*	NFCCI 3048	5.64 ± 0.12	2.00 ± 0.44	7.39 ± 0.24
*Fusarium annulatum*	NFCCI 3300	7.10 ± 0.34	0.66 ± 0.05	7.62 ± 0.54
*Fusarium annulatum*	NFCCI 2964	7.07 ± 0.19	1.11 ± 0.18	7.5 ± 0.13
*Fusarium mangiferae*	NFCCI 2885	7.97 ± 0.28	-	7.8 ± 0.15
*Fusarium grosmichelii*	NFCCI 3243	6.37 ± 0.59	1.44 ± 0.083	7.57 ± 0.21
*Fusarium annulatum*	NFCCI 2962	7.42 ± 0.06	3.26 ± 0.23	7.49 ± 0.17
*Fusarium nanum*	NFCCI 5192	6.40 ± 0.18	-	7 ± 0.14
*Fusarium sacchari*	NFCCI 3093	8.61 ± 0.83	2.31 ± 0.58	7.32 ± 0.22
*Fusarium sulawesiense*	NFCCI 2956	6.44 ± 0.37	-	7.44 ± 0.29
*Neocosmospora metavorans*	NFCCI 5193	6.25 ± 0.46	-	7.34 ± 0.17
*Fusarium sulawesiense*	NFCCI 2886	5.89 ± 0.27	-	7.34 ± 0.12
*Fusarium annulatum*	NFCCI 2959	6.13 ± 0.49	-	7.29 ±0.18
*Fusarium sulawesiense*	NFCCI 3031	8.99 ± 0.67	-	7.31 ± 0.48
*Fusarium compactum*	NFCCI 2946	8.89 ± 0.11	0.08 ± 0.04	7.44 ± 0.42
*Neocosmospora vasinfecta*	NFCCI 2960	6.64 ± 0.41	4.11 ± 0.17	7.44 ± 0.13
*Fusarium brachygibbosum*	NFCCI 3703	5.74 ± 0.32	0.15 ± 0.06	8.79 ± 0.04
*Fusarium irregulare*	NFCCI 5194	6.58 ± 0.48	1.88 ± 0.06	8.5 ± 0.20
*Neocosmospora oblonga*	NFCCI 2150	5.06 ± 0.19	-	8.67 ± 0.37
*Neocosmospora metavorans*	NFCCI 3475	6.51 ± 0.22	-	8.64 ± 0.06
*Fusarium commune*	NFCCI 2871	8.15 ± 0.18	-	8.67 ± 0.18
*Neocosmospora metavorans*	NFCCI 4095	6.71 ± 0.37	-	8.96 ± 0.28
*Fusarium spinosum*	NFCCI 5195	9.15 ± 0.64	-	8.9 ± 0.07
*Fusarium microconidium*	NFCCI 3020	8.52 ± 0.13	-	8.82 ± 0.08
*Fusarium cugenangense*	NFCCI 2872	7.01 ± 0.07	4.47 ± 0.51	8.69 ± 0.15
*Fusarium irregulare*	NFCCI 2460	9.04 ± 0.13	-	8.84 ± 0.03
*Fusarium annulatum*	NFCCI 3065	6.22 ± 0.42	0.20 ± 0.06	8.93 ± 0.17
*Fusarium tardicrescens*	NFCCI 680	7.12 ± 0.34	0.47 ± 0.07	8.87 ± 0.05
*Fusarium sacchari*	NFCCI 5196	9.18 ± 0.18	0.60 ± 0.06	7.87 ± 0.05
*Fusarium lacertarum*	NFCCI 5197	7.79 ± 2.1	0.49 ± 0.17	8.03 ± 0.02
*Fusarium irregulare*	NFCCI 5198	11.30 ± 4.8	-	8.36 ± 0.15
*Fusarium annulatum*	NFCCI 2470	10.71 ± 0.29	-	7.87 ± 0.13
*Fusarium sacchari*	NFCCI 3091	5.68 ± 0.17	-	7.28 ± 0.05
*Neocosmospora metavorans*	NFCCI 5199	5.35 ± 0.57	-	7.42 ± 0.17
*Fusarium brachygibbosum*	NFCCI 3074	7.80 ± 0.69	0.01 ± 0.05	7.72 ± 0.21
*Neocosmospora suttoniana*	NFCCI 2961	10.24 ± 0.7	0.67 ± 0.07	8.17 ± 0.45
*Fusarium lacertarum*	NFCCI 3049	7.22 ± 0.51	2.39 ± 0.39	7.19 ± 0.16
*Fusarium sacchari*	NFCCI 3092	5.79 ± 0.14	-	7.26 ± 0.15
*Fusarium annulatum*	NFCCI 1127	5.56 ± 0.61	-	6.92 ± 0.09
*Fusarium fabacearum*	NFCCI 3706	7.30 ± 0.47	1.00 ± 0.20	8.6 ± 0.05
*Fusarium compactum*	NFCCI 2904	6.45 ± 0.18	3.33 ± 0.51	7.02 ± 0.04
*Fusarium lacertarum*	NFCCI 3038	7.59 ± 0.09	0.16 ± 0.06	8.07 ± 0.15
*Fusarium annulatum*	NFCCI 2953	7.38 ± 0.15	2.21 ± 0.12	7.85 ± 0.17
*Fusarium tardichlamydosporum*	NFCCI 3051	7.99 ± 0.12	2.19 ± 0.18	7.92 ± 0.13
*Fusarium duoseptatum*	NFCCI 681	6.37 ± 0.28	-	6.86 ± 0.12
*Fusarium annulatum*	NFCCI 3270	5.14 ± 0.19	4.84 ± 0.36	7.24 ± 0.17
*Fusarium verticillioides*	NFCCI 2945	5.43 ± 1.2	-	7.18 ±0.3
*Neocosmospora vasinfecta*	NFCCI 2972	7.61 ± 2.1	-	7.32 ± 0.02
*Fusarium annulatum*	NFCCI 2053	4.64 ± 0.09	3.78 ± 0.55	7.73 ± 0.5
*Fusarium proliferatum*	NFCCI 3282	6.51 ± 3.2	0.16 ± 0.19	7.55 ± 0.12
*Fusarium fabacearum*	NFCCI 5200	10.07 ± 0.17	14.25 ± 0.28	8.11 ± 0.16
*Fusarium tardicrescens*	NFCCI 5201	11.23 ± 0.38	15.82 ± 0.54	8.2 ± 0.11
*Fusarium sacchari*	NFCCI 4889	14.17 ± 2.7	5.95 ± 0.61	8.23 ± 0.04
*Albonectria rigidiuscula*	NFCCI 5202	14.03 ± 0.07	0.16 ± 0.04	7.39 ± 0.13
*Fusarium tardichlamydosporum*	NFCCI 1895	7.80 ± 1.8	0.12 ± 0.05	7.27 ± 0.24
*Fusarium pernambucanum*	NFCCI 5203	9.90 ± 0.15	1.04 ± 0.11	8.09 ± 0.15
*Fusarium carminascens*	NFCCI 5204	11.15 ± 2.4	13.54 ± 0.62	8.44 ± 0.19
*Fusarium nirenbergiae*	NFCCI 4859	6.52 ± 0.08	-	7.5 ± 0.32
*Fusarium irregulare*	NFCCI 5205	9.32 ± 1.6	-	7.59 ± 0.27
*Fusarium brachygibbosum*	NFCCI 4972	11.03 ± 0.15	-	7.66 ± 0.21
*Fusarium brachygibbosum*	NFCCI 5206	8.52 ± 2.9	-	7.66 ± 0.51
*Fusarium pernambucanum*	NFCCI 5207	9.80 ± 2.6	1.43 ± 0.16	7.22 ± 0.18
*Fusarium oxysporum*	NFCCI 4759	7.69 ± 0.14	2.22 ± 0.43	7.12 ± 0.16
*Fusarium verticillioides*	NFCCI 4963	10.55 ± 3.7	-	7.55 ± 0.14
*Fusarium verticillioides*	NFCCI 2696	11.05 ± 2.7	-	7.85 ± 0.05
*Fusarium compactum*	NFCCI 5208	4.41 ± 0.13	1.146 ± 0.13	6.67 ± 0.07
*Fusarium gossypinum*	NFCCI 2467	4.59 ± 0.17	0.27 ± 0.08	8.29 ± 1.5
*Fusarium acutatum*	NFCCI 5209	4.69 ± 0.15	0.29 ± 0.06	6.38 ± 0.7
*Neocosmospora suttoniana*	NFCCI 5210	7.50 ± 3.7	0.90 ± 0.08	7.86 ± 0.36
*Neocosmospora metavorans*	NFCCI 4885	7.12 ± 2.5	0.86 ± 0.07	7.94 ± 0.59
*Fusarium glycines*	NFCCI 1788	11.20 ± 0.13	1.05 ± 0.10	8.04 ± 0.16
*Fusarium tardichlamydosporum*	NFCCI 2491	13.59 ± 0.19	1.38 ± 0.11	8 ± 0.08
*Albonectria rigidiuscula*	NFCCI 4888	13.77 ± 0.27	1.09 ± 0.08	8.13 ± 0.8
*Neocosmospora suttoniana*	NFCCI 4830	4.67 ± 2.6	1.01 ± 0.05	7.65 ±0.02
*Neocosmospora suttoniana*	NFCCI 5211	8.56 ± 3.1	-	8 ± 0.07
*Fusarium annulatum*	NFCCI 5212	10.94 ± 1.2	-	8.14 ± 0.4
*Fusarium lumajangense*	NFCCI 4180	8.26 ± 2.7	1.07 ± 0.39	8.18 ±0.05
*Fusarium lacertarum*	NFCCI 4792	9.66 ± 1.8	-	8.23 ± 0.1
*Fusarium annulatum*	NFCCI 2949	6.70 ± 0.27	-	8.16 ± 0.02
*Fusarium sulawesiense*	NFCCI 4919	8.72 ± 0.44	-	8.25 ± 0.14

- Not Detected; All values represent means ± S.E.M.

## Data Availability

Not applicable.

## Supplementary Materials

The following supporting information can be downloaded at https://www.mdpi.com/article/10.3390/jof8070662/s1, Figure S1: Fermentation of different isolates in *Fusarium* defined media after a week’s fermentation (a) Control (Uninnoculated FDM medium) (b) *Fusarium fabacearum* NFCCI 5200 (c) *F. annulatum* NFCCI 3300 (d) *F. sulawesiense* NFCCI 4919 (e) *F. sacchari* NFCCI 4889 (f) *F. annulatum* NFCCI 5212 (g) *F. nirenbergiae* NFCCI 5189 (h) *F. tardicrescens* NFCCI 5201 (i) *F. lacertarum* NFCCI 4792 (j) *F. lumajangense* NFCCI 4180 (k) *F. pernambucanum* NFCCI 5203 (l) *F. duoseptatum* NFCCI 681 (m) *F. tardichlamydosporum* NFCCI 1895 (n) *Neocosmospora solani* NFCCI 2315 (o) *F. mangiferae* NFCCI 2885 (p) *F. annulatum* NFCCI 2964 (q) *F. sacchari* NFCCI 3147 (r) *F. grosmichelii* NFCCI 3243; Table S1: GenBank Accession No. and other details of the isolates used in the phylogenetic study.
