# Supplementary material for: Multigene Phylogeny, Beauvericin Production and Bioactive Potential of Fusarium Strains Isolated in India
Source: J Fungi (Basel). 2022 Jun 24;8(7):662. doi: 10.3390/jof8070662 (PMC9320867; doi:10.3390/jof8070662)
Supplement: Supplementary file 1 [file jof-08-00662-s001.zip › jof-1752460-supplementary.pdf]

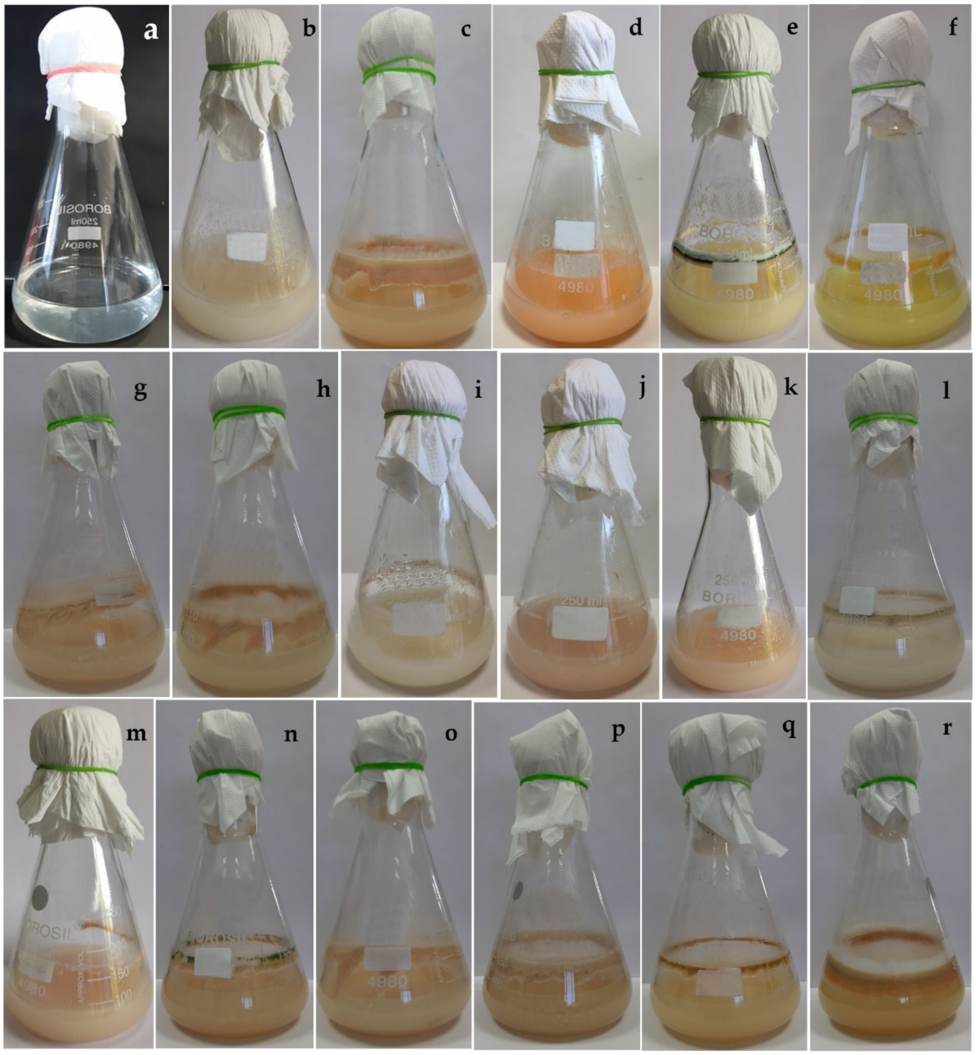

Figure S1. Fermentation of different isolates in *Fusarium* defined media after a week's fermentation (a) Control (Uninnoculated FDM medium); (b) *Fusarium fabacearum* NFCCI 5200; (c) *F. annulatum* NFCCI 3300; (d) *F. sulawesiense* NFCCI 4919; (e) *F. sacchari* NFCCI 4889; (f) *F. annulatum* NFCCI 5212; (g) *F. nirenbergiae* NFCCI 5189; (h) *F. tardicrescens* NFCCI 5201 (i) *F. lacertarum* NFCCI 4792; (j) *F. lumajangense* NFCCI 4180 (k) *F. pernambucanum* NFCCI 5203; (l) *F. duoseptatum* NFCCI 681; (m) *F. tardichlamydosporum* NFCCI 1895; (n) *Neocosmospora solani* NFCCI 2315; (o) *F. mangiferae* NFCCI 2885; (p) *F. annulatum* NFCCI 2964; (q) *F. sacchari* NFCCI 3147; (r) *F. grosmichelii* NFCCI 3243

Table S1. GenBank Accession No. and other details of the isolates used in the phylogenetic study

Isolate	Culture	Locality	Substrate	*Rpb2*	*Tef1-α*	ITS	LSU	*β-tub*	*CaM*
Albonectria rigidiuscula	CBS 122570			HQ897760	KM231937	HQ897815	KM231676	KM232070	KM231382
Albonectria rigidiuscula	**NFCCI 5202**	India, Pilicode, Kasaragod, Kerala	Cashew wilt	ON398234	ON032442	ON003547	OM837261		OM960794
Albonectria rigidiuscula	**NFCCI 4888**	India, Jobner, Jaipur, Rajasthan	Cucumber twig	ON398253	ON032461	ON003566	OM837280		-
Atractium stilbaster	CBS 410.67	Germany	Decaying bark		KM231920	MH859011	MH870712	KM232050	KM231357
F. tardichlamydosporum	**NFCCI 2491**	India, Bangalore, Karnataka	Soil	ON398252	ON032460	ON003565	OM837279		OM960808
Fusarium aberrans	CBS 131385	Australia, Northern Territory	Stem of *Oryza australiensis*	MN170378	MN170445				MN170311
Fusarium acutatum	BBA 69580 = CBS 402.97 = FRC 0-1117 = IMI 376110 = NRRL 13309	India	Unknown	MW402768	MW402125	MH862652	MH874259		MW402459
Fusarium acutatum	**NFCCI 5209**	India, Pithoragarh, Uttarakhand	Carrot rhizosphere	ON398248	ON032456	ON003561	OM837275	OM960822	OM960804
Fusarium annulatum	BBA 63629 = CBS 258.54 = IMI 202878 = MUCL 8059 = NRRL 13619	New Caledonia	Grain of *Oryza sativa*	MT010983	MT010994	MH857317	MH868857	MT011041	MT010908
Fusarium annulatum	**NFCCI 3264**	India, Thiruvananthapura, Kerala	Cow pea	ON398174	ON032382	ON003487	OM837201		
Fusarium annulatum	**NFCCI 3072**	India, Maharashtra	*Barleria prionitis* stem	ON398181	ON032389	ON003494	OM837208		OM960748
Fusarium annulatum	**NFCCI 3300**	India, Raipur, Chhattisgarh	*Cathranthus roseus*	ON398183	ON032391	ON003496	OM837210		OM960750
Fusarium annulatum	**NFCCI 2964**	India, Latur, Maharashtra	Pigeon pea root	ON398184	ON032392	ON003497	OM837211		OM960751
Fusarium annulatum	**NFCCI 2962**	India, Kalamb, Osmanabad, Maharashtra	Pigeon pea root	ON398187	ON032395	ON003500	OM837214		OM960754
Fusarium annulatum	**NFCCI 2959**	India, Amravati, Maharashtra	Pigeon pea root	ON398193	ON032401	ON003506	OM837220		OM960760
Fusarium annulatum	**NFCCI 3065**	India, Maharashtra	*Ocimum sanitum*	ON398207	ON032415	ON003520	OM837234		OM960773

Fusarium annulatum	**NFCCI 2470**	India, Vellayani, Thiruvananthapuram, Kerala	Coconut leaves	ON398212	ON032420	ON003525	OM837239		OM960776
Fusarium annulatum	**NFCCI 1127**	India, Durg, Chhattisgarh	*Jatropa curcus* (phyloplane)	ON398219	ON032427	ON003532	OM837246		OM960782
Fusarium annulatum	**NFCCI 2953**	India, Shirpur, Maharashtra	Cotton field	ON398223	ON032431	ON003536	OM837250		OM960785
Fusarium annulatum	**NFCCI 3270**	India, Mumbai, Maharashtra	Dead wood	ON398226	ON032434	ON003539	OM837253		OM960788
Fusarium annulatum	**NFCCI 2053**	India, Faizabad, Rajasthan	Wilted Guava plant (root)	ON398229	ON032437	ON003542	OM837256		
Fusarium annulatum	**NFCCI 5212**	India, Nashik, Maharashtra	Grape leaf	ON398256	ON032464	ON003569	OM837283		
Fusarium annulatum	**NFCCI 2949**	India, Shirpur, Maharashtra	Cotton field	ON398259	ON032467	ON003572	OM837286		OM960811
Fusarium arcuatisporum	CGMCC 3.19493 = LC 12147	China, Hubei	Pollen of *Brassica campestris*	MK289739	MK289584	MK280802			MK289697
Fusarium brachiariae	CML 3032	Brazil, Mato Campo Grande	Seed of *Brachiaria decumbens*	MT901314	MT901348			MT901321	
Fusarium brachygibbosum	BBA 64691 = NRRL 20954	India, Telangana, Parbhani	*Sorghum vulgare*	MW233418	MW233075				
Fusarium brachygibbosum	**NFCCI 3703**	India, Nanded, Maharashtra	Onion	ON398197	ON032405	ON003510	OM837224		OM960763
Fusarium brachygibbosum	**NFCCI 3074**	India, Maharashtra	*Barleria prionitis*	ON398215	ON032423	ON003528	OM837242		OM960778
Fusarium brachygibbosum	**NFCCI 4972**	India, Dharur, Beed, Maharashtra	Sugarcane baggase	ON398240	ON032448	ON003553	OM837267		
Fusarium brachygibbosum	**NFCCI 5206**	India, Vadodara, Gujarat	Soil	ON398241	ON032449	ON003554	OM837268		
Fusarium brevicaudatum	NRRL 43638 = UTHSC R-3500	USA, Florida	*Trichechus* sp.	GQ505843	GQ505665	GQ505754			GQ505576
Fusarium caatingaense	MUM 1859 = URM 6779	Brazil, Pernambuco, Ibimirim	*Dactylopius opuntiae*	LS398495	LS398466	MH668816	MH307669		
Fusarium caatingaense	**NFCCI 5191**	India, Pune, Maharashtra	Pomegranate	ON398179	ON032387	ON003492	OM837206		OM960746

Fusarium callistephi	CBS 187.53 = NRRL 36330	Netherlands, Oostenbrink	*Callistephus chinensis*	MH484875	MH484966			MH485057	MH484693
Fusarium carminascens	CBS 144738 = CPC 25800	South Africa, KwaZulu-Natal Province	*Zea mays*	MH484937	MH485028		MH484846	MH485119	MH484755
Fusarium carminascens	**NFCCI 5204**	India, Pune, Maharashtra	Potato	ON398237	ON032445	ON003550	OM837264		
Fusarium clavus	CBS 126202 = RMF N 38	Namibia, northern Karoo	Desert soil	MN170389	MN170456	MH864013	MH875469		MN170322
Fusarium commune	AAS 156 = BBA 71639 = CBS 110090 = NRRL 31076	Denmark.	Soil	MW934368	AF362263				
Fusarium commune	**NFCCI 2871**	India, Manipur	Wilted tomato plant (root)	ON398201	ON032409	ON003514	OM837228		OM960767
Fusarium compactum	CBS 186.31 = NRRL 36323	UK, Kew.	Cotton thread	GQ505826	GQ505648	GQ505737			GQ505560
Fusarium compactum	**NFCCI 2946**	India, Karnataka	*Aegla marmelos*	ON398195	ON032403	ON003508	OM837222		
Fusarium compactum	**NFCCI 2904**	India, Karnataka	*Aegla marmelos*	ON398221	ON032429	ON003534	OM837248		
Fusarium compactum	**NFCCI 5208**	India, Aurangabad, Maharastra	*Zingiber officinale*	ON398246	ON032454	ON003559	OM837273		OM960802
Fusarium concentricum	BBA 64354 = CBS 450.97 = DAOM 225146 = IMI 375352 = NRRL 25181	Costa Rica	*Musa sapientum*	LT575063	AF160282	NR_111886		MT011040	MT010906
Fusarium contaminatum	CBS 114899	Germany, Schlüchtern	Pasteurised chocolate milk	MH484901	MH484992			MH485083	MH484719
Fusarium cugenangense	Indo203	Indonesia, West Java, Cugenang	Pseudostem of *Musa* var. *Pisang Kepok*	LS479308	LS479757				
Fusarium cugenangense	**NFCCI 2872**	India, Manipur	Wilted tomato plant (root)	ON398205	ON032413	ON003518	OM837232		OM960771
Fusarium curvatum	CBS 238.94 = NRRL 26422 = PD 94/184	Netherlands	*Beaucarnea* sp.	MH484893	MH484984			MH485075	MH484711
Fusarium duofalcatisporum	CBS 384.92 = NRRL 36448	Sudan, Nile Province	Seeds of *Phaseolus vulgaris*	GQ505830	GQ505652	GQ505741			GQ505564
Fusarium duoseptatum	InaCC F916/ Indo109	Indonesia	Pseudostem of *Musa* var. *Pisang Kepok*	LS479239	LS479688				

Fusarium duoseptatum	**NFCCI 681**	India, Maharashtra	*Azadirachta indica* (Endophyte)	ON398225	ON032433	ON003538	OM837252	OM960817	OM960787
Fusarium elaeidis	CBS 217.49 = NRRL 363580	Zaire	*Elaeis* sp.	MH484870	MH484961			MH485052	MH484688
Fusarium fabacearum	CBS 144743 = CPC 25802	South Africa, Western Cape Province	*Glycine max*	MH484938	MH485029			MH485121	MH484757
Fusarium fabacearum	**NFCCI 3239**	India, Mumbai, Maharashtra	Marigold seeds	ON398177	ON032385	ON003490	OM837204	OM960813	OM960744
Fusarium fabacearum	**NFCCI 3706**	India, Aanand, Gujarat	Castor root	ON398220	ON032428	ON003533	OM837247		OM960783
Fusarium fabacearum	**NFCCI 5200**	India, Pune, Maharashtra	Pea	ON398231	ON032439	ON003544	OM837258	OM960818	OM960791
Fusarium foetens	CBS 110286 = NRRL 31852 = PD 2001/7244	Netherlands	*Begonia elatior* hybrid	MW928825	AY320087	MH862858	MH874448	MH485121	MH484757
Fusarium fujikuroi	BBA 12428 = BBA 63630 = CBS 221.76 = IHEM 3821 = IMI 196086 = IMI 202879 = NRRL 13620 = NRRL 13998 = NRRL 22174	Taiwan	*Oryza sativa*	JX171570	AF160279	MW827608	MW827648	MN534130	KU603995
Fusarium globosum	CBS 428.97 = DOAM 214966 = FRC M8014 = IMI 375330 = MRC 6647 = NRRL 26131 = PREM 51878	South Africa, Eastern Cape Province	*Zea mays*	KF466406	KF466417			MT011042	MT010907
Fusarium glycines	CBS 144746 = CPC 25808	South Africa, North West Province	*Glycine max*	MH484942	MH485033			MH485124	MH484760
Fusarium glycines	**NFCCI 3048**	India, Gujarat	Wilted cumin plant	ON398182	ON032390	ON003495	OM837209		OM960749
Fusarium glycines	**NFCCI 1788**	India, Imphal, Manipur	Soil	ON398251	ON032459	ON003564	OM837278		OM960807
Fusarium gossypinum	CBS 116613	Ivory Coast	*Gossypium hirsutum*	MH484909	MH485000			MH485091	MH484727
Fusarium gossypinum	**NFCCI 2467**	India, Dapoli, Maharastra	Bottle Gourd	ON398247	ON032455	ON003560	OM837274		OM960803
Fusarium grosmichelii	JBR A	Indonesia, West Java	Pseudostem of *Musa acuminata* var. *Pisang*	LS479295	LS479744				

			Ambon Lumut						
Fusarium grosmichelii	**NFCCI 3243**	India, Thiruvananthapuram, Kerala	Cow pea	ON398186	ON032394	ON003499	OM837213		OM960753
Fusarium hainanense	CGMCC 3.19478 = LC11638	China, Hainan Province	Stem of *Oryza* sp.	MK289735	MK289581	MK280836			MK289657
Fusarium hexaseptatum	Indo55	Indonesia, West Java, Sukabumi, Parakan Lima.	*Musa acuminata* var. *Pisang Ambon Kuning*	LS479359	LS479805				
Fusarium hoodiae	CBS 132474	South Africa, Northern Cape Province, Prieska	Root of *Hoodia gordonii*	MH484929	MH485020	MH866022	MH877470	MH485111	MH484747
Fusarium incarnatum	ATCC 24387 = CBS 132.73 = IMI 128222 = NRRL 25478	Malawi	*Trichosanthes dioica*	MN170409	MN170476		MH878482		MN170342
Fusarium inflexum	ATCC 32213 = BBA 63203 = CBS 716.74 = DAOM 225130 = DSM 63203 = IMI 375336 = NRRL 20433	Germany, Hamburg, Vierlanden	Stem of *Vicia faba*	JX171583	AF008479	NR_152941	U34548		
Fusarium ipomoeae	CGMCC 3. 19496 = LC12165	China, Jiangsu Province	Leaves of *Ipomoea aquatica*	MK289752	MK289599	MK280832			MK289704
Fusarium irregulare	CGMCC 3.19489 = LC7188	China, Guangdong Province	*Bambusoideae*	MK289863	MK289629	MK280829			MK289680
Fusarium irregulare	**NFCCI 5194**	India, Mahabaleshwar, Maharashtra	Forest litter	ON398198	ON032406	ON003511	OM837225		OM960764
Fusarium irregulare	**NFCCI 2460**	India, Udhampur, Jammu and Kashmir	*Zanthooxylum armatum*	ON398206	ON032414	ON003519	OM837233		OM960772
Fusarium irregulare	**NFCCI 5198**	India, Pune, Maharashtra	Soil	ON398211	ON032419	ON003524	OM837238		
Fusarium irregulare	**NFCCI 5205**	India, Pune, Maharashtra	Aloe vera leaf	ON398239	ON032447	ON003552	OM837266		OM960797

Fusarium kalimantanense	Indo110	Indonesia, Central Kalimantan, Katingan, Pulau Malan	*Musa acuminata* var. *Pisang Ambon*	LS479241	LS479690				
Fusarium lacertarum	ATCC 42771 = CBS 130185 = IMI 300797 = NRRL 20423	India, Poona, Pimpri	Skin of lizard	JX171581	GQ505593	GQ505682			GQ505505
Fusarium lacertarum	**NFCCI 3044**	India, Gujarat	Wilted cumin plant	ON398175	ON032383	ON003488	OM837202		OM960743
Fusarium lacertarum	**NFCCI 5197**	India, Mahabaleshwar, Maharashtra	*Crysanthomum roseus*	ON398210	ON032418	ON003523	OM837237		
Fusarium lacertarum	**NFCCI 3049**	India, Gujarat	Wilted cumin plant	ON398217	ON032425	ON003530	OM837244		OM960780
Fusarium lacertarum	**NFCCI 3038**	India, Gujarat	Wilted cumin plant	ON398222	ON032430	ON003535	OM837249		OM960784
Fusarium lacertarum	**NFCCI 4792**	India, Pratapgarh, Uttar Pradesh	Chickpea root	ON398258	ON032466	ON003571	OM837285		OM960810
Fusarium languescens	CBS 645.78 = NRRL 36531	Morocco	*Solanum lycopersicum*	MH484880	MH484971			MH485062	MH484698
Fusarium libertatis	CBS 144749 = CPC 28465	South Africa, Western Cape Province	Rock surface	MH484944	MH485035			MH485126	MH484762
Fusarium longicaudatum	ATCC 24370 = CBS 123.73 = IMI 160825 = NRRL 25477	Tanzania, Tropical Products Research Inst.	Unknown	MN170414	MN170481				MN170347
Fusarium luffae	CGMCC 3.19497 = LC12167	China, Fujian	*Luffa aegyptiaca*	MK289754	MK289601	MK280807			MK289698
Fusarium lumajangense	InaCC F872	Indonesia, East Java, Lumajang	*Musa acuminata* var. *Pisang Mas Kirana*	LS479850	LS479441			LS479433	
Fusarium lumajangense	**NFCCI 4180**	India, Chandigarh	Soil	ON398257	ON032465	ON003570	OM837284		
Fusarium mangiferae	CBS 120994 = KSU 11781 = MRC 7559 = MUCL 54671 = NRRL 53980	Israel, Bet Dagan	*Mangifera indica*	LT575059	LT574978			MN534128	MN534224

Fusarium mangiferae	**NFCCI 2885**	India, Chandravati, Rajasthan	*Azadirachta indica* (Endophyte)	ON398185	ON032393	ON003498	OM837212	OM960814	OM960752
Fusarium microconidium	CBS 119843 = MRC 8391 = KSU 11396	Unknown	Unknown		MN120759		MH874619		MN120700
Fusarium microconidium	**NFCCI 3020**	India, Karnataka	Soil	ON398204	ON032412	ON003517	OM837231		OM960770
Fusarium monophialidicum	NRRL 54973 = UTHSC 06-1473	USA, Ohio	Eye of *Rhinocerotidae*	KC808362	MN170483				MN170349
Fusarium multiceps	CBS 130386 = NRRL 43639 = UTHSC 04-135	USA, Florida	*Trichechus* sp.	GQ505844	GQ505666	GQ505755			GQ505577
Fusarium musae	CBS 624.87 = MUCL 52574 = NRRL 25059	Honduras	*Musa* sp.	FN552108	FN552086			FN545368	MW402474
Fusarium nanum	CGMCC 3.19498 = LC12168	China, Guangxi Province	Leaves of *Musa nana*	MK289755	MK289602	MK280794			MK289651
Fusarium nanum	**NFCCI 5192**	India, Aurangabad, Maharashtra	*Zingiber officinale*	ON398188	ON032396	ON003501	OM837215		OM960755
Fusarium nelsonii	CBS 119876 = FRC R-8670 = MRC 4570 = NRRL 28505 = NRRL 53945	South Africa, Western Cape Province	Plant debris in *Triticum* soil	GQ505468	GQ505404	GQ505436			MN120701
Fusarium nirenbergiae	CBS 840.88	Netherlands, Noord-Holland Province	*Dianthus caryophyllus*	MH484887	MH484978			MH485069	MH484705
Fusarium nirenbergiae	**NFCCI 5189**	India, Haridwar, Uttarakhand	Soil	ON398173	ON032381	ON003486	OM837200	OM960812	OM960742
Fusarium nirenbergiae	**NFCCI 4859**	India, Arunachal Pradesh	*Dillenia indica*	ON398238	ON032446	ON003551	OM837265	OM960821	OM960796
Fusarium odoratissimum	InaCC F822/ Indo8	Indonesia, East Kalimantan	*Musa* sp. cv. Pisang Kepok	LS479386	LS479828				
Fusarium oxysporum	CBS 144134	Germany, Berlin	*Solanum tuberosum*	MH484953	MH485044			MH485135	MH484771
Fusarium oxysporum	**NFCCI 4759**	India, Imphal, Manipur	*Anaphalis contorta* root (Endophyte)	ON398243	ON032451	ON003556	OM837270		OM960799

Fusarium pernambucanum	MUM 1862 = URM 7559	Brazil, Pernambuco	*Aleurocanthus woglumi*	LS398519	LS398489	NR_163754	MH307693		
Fusarium pernambucanum	**NFCCI 5203**	India, Simbal, Himachal Pradesh	Rotting mushroom	ON398236	ON032444	ON003549	OM837263		OM960795
Fusarium pernambucanum	**NFCCI 5207**	India, Goa	Coconut	ON398242	ON032450	ON003555	OM837269		OM960798
Fusarium persicinum	CBS 479.83	Unknown	Unknown	MN170428	MN170495				MN170361
Fusarium pharetrum	CBS 144751 = CPC 30824	South Africa	*Aloidendron dichotomum*	MH484952	MH485043			MH485134	MH484770
Fusarium phialophorum	InaCC F971/ Indo182	Indonesia, South Kalimantan	*Musa* var. *Pisang Awak*	LS479292	LS479741				
Fusarium proliferatum	CBS 480.96 = IAM 14682 = NRRL 26427 = NY007.B6	Papua New Guinea	Forest soil	MN534272	MN534059			MN534129	MN534217
Fusarium proliferatum	**NFCCI 3282**	India, Amarkantak, Madhya Pradesh	Soil	ON398230	ON032438	ON003543	OM837257		OM960790
Fusarium sacchari	BBA 63340 = CBS 223.76 = DAOM 225138 = IMI 202881 = NRRL 13999	India	*Saccharum officinarum*	JX171580	AF160278			MW402313	KU604012
Fusarium sacchari	**NFCCI 3147**	India, Dapoli, Maharashtra	Red rotted sugarcane	ON398178	ON032386	ON003491	OM837205		OM960745
Fusarium sacchari	**NFCCI 3093**	India, Tamilnadu	Textile sludge	ON398189	ON032397	ON003502	OM837216		OM960756
Fusarium sacchari	**NFCCI 5196**	India, Gorakhpur, Uttar Pradesh	Wilted sugarcane plant	ON398209	ON032417	ON003522	OM837236		OM960775
Fusarium sacchari	**NFCCI 3091**	India, Tamilnadu	Textile sludge	ON398213	ON032421	ON003526	OM837240		OM960777
Fusarium sacchari	**NFCCI 3092**	India, Tamilnadu	Textile sludge	ON398218	ON032426	ON003531	OM837245		OM960781
Fusarium sacchari	**NFCCI 4889**	India, Vishakhapatnum Andhra pradesh	Unknown insect	ON398233	ON032441	ON003546	OM837260		OM960793
Fusarium sangayamense	InaCC F960/ Indo165	Indonesia, South Kalimantan, Kota Baru	Pseudostem of *Musa* var. *Pisang Kepok*	LS479283	LS479732				
Fusarium siculi	CBS 142422 = CPC 27188	Italy, Sicily	*Citrus sinensis*	LT746327	LT746214	LT746262		LT746346	LT746189
Fusarium spinosum	CBS 122438	Brazil	*Galia melon*	MN120747	MN120768				MN120708

Fusarium spinosum	**NFCCI 5195**	India, Chandigarh	Soil	ON398203	ON032411	ON003516	OM837230		OM960769
Fusarium sulawesiense	Indo138	Indonesia, South Sulawesi Bengo, Desa Selli	Infected pseudostem of *Musa acuminata* var. *Pisang Cere* (AAA)	LS479855	LS479443	LS479410	LS479883		LS479422
Fusarium sulawesiense	**NFCCI 2956**	India, Shirpur, Maharashtra	Cotton field	ON398190	ON032398	ON003503	OM837217		OM960757
Fusarium sulawesiense	**NFCCI 2886**	India, Chikkamagaluru, Karnataka	*Azadirachta indica* (Endophyte)	ON398192	ON032400	ON003505	OM837219		OM960759
Fusarium sulawesiense	**NFCCI 3031**	India, Tamilnadu	Piberbettle leaf (endophyte)	ON398194	ON032402	ON003507	OM837221		OM960761
Fusarium sulawesiense	**NFCCI 4919**	India, Pathanamthitta, Kerala	Soil	ON398260	ON032468	ON003573	OM837287		
Fusarium tardichlamydosporum	InaCC F958	Indonesia, East Nusa Tenggara	Pseudostem of *Musa acuminata* var. *Pisang Barangan*	LS479280	LS479729				
Fusarium tardichlamydosporum	**NFCCI 3051**	India, Gujarat	Wilted cumin plant	ON398224	ON032432	ON003537	OM837251	OM960816	OM960786
Fusarium tardichlamydosporum	**NFCCI 1895**	India, Mahabaleshwar, Maharashtra	Soil	ON398235	ON032443	ON003548	OM837262	OM960820	
Fusarium tardicrescens	CBS 102024 = NRRL 36113	Malawi, Karonga, Misuku Hills	*Musa sapientum* cv. *Harare*	LS479217	LS479665				
Fusarium tardicrescens	**NFCCI 680**	India, Maharashtra	*Azadirachta indica* (Endophyte)	ON398208	ON032416	ON003521	OM837235	OM960815	OM960774
Fusarium tardicrescens	**NFCCI 5201**	India, Simbal, Himachal Pradesh	Dead bark	ON398232	ON032440	ON003545	OM837259	OM960819	OM960792
Fusarium transvaalense	CBS 144211	South Africa, Kruger National Park, Skukuza, Granite Supersite	Rhizosphere of *Sida cordifolia*	LT996157	LT996099			LT996120	
Fusarium triseptatum	CBS 258.50 = NRRL 36389	USA	*Ipomoea batatas*	MH484873	MH484964			MH485055	MH484691

Fusarium verticillioides	BBA 11782 = CBS 218.76 = DSM 62264 = IMI 202875 = NRRL 13993	Germany	*Zea mays*	MW928835	KF499582			MW402311	MW402449
Fusarium verticillioides	**NFCCI 2945**	India, Tiruchirappalli, Tamil Nadu	Soil	ON398227	ON032435	ON003540	OM837254		
Fusarium verticillioides	**NFCCI 4963**	India, Raiganj, Uttar dinajpur, West Bengal	Plant Litter	ON398244	ON032452	ON003557	OM837271		OM960800
Fusarium verticillioides	**NFCCI 2696**	India, Puthenthope, Kerala	*Coleus* sp.	ON398245	ON032453	ON003558	OM837272		OM960801
Fusarium veterinarium	CBS 109898 = NRRL 36153	Netherlands	Peritoneum of *Selachimorpha*	MH484899	MH484990			MH485081	MH484717
Geejayessia cicatricum	CBS 125549	Slovenia, Arboretum	Decaying twigs of *Buxus sempervirens*	HM626679	HM626643	MH863558	MH875036	KM232073	KM231385
Geejayessia zealandica	BBA 64792 = CBS 111.93	New Zealand, Auckland	Bark of *Hoheria populnea*	HM626684	HQ728148	NR_138298	NG_060389		
Neocosmospora cyanescens	CBS 518.82T	Netherlands	Human foot	LR583826	LR583605	AB190389	AB190419		MW218064
Neocosmospora cyanescens	CBS 637.82	Netherlands	Human foot	LR583827	LR583606	LR583712	LR583921		MW218065
Neocosmospora falciformis	CBS 121450	Syria	Grape vine	JX435261	JX435161	JX435211	JX435211		MW218069
Neocosmospora falciformis	NRRL 43529 = CDC 2006743575	USA	Human cornea	JX171653	EF452965	EF453117	EF453117		
Neocosmospora ferruginea	CBS 109028	Switzerland	Human subcutaneous nodule	EU329581	DQ246979	DQ094446	DQ236488		MW834132
Neocosmospora ferruginea	CPC 28194	Italy	*Citrus sinensis*	LT746341	LR583602	LT746276	LT746276		MW834133
Neocosmospora ferruginea	NRRL 32437	Switzerland	Subcutaneous nodule of *Homo sapiens*	EU329581	DQ246979	JX435216	DQ236488		MW834132
Neocosmospora longissima	CBS 126407 G.J.S. 85-72	New Zealand, Russell State Forest	From tree bark	LR583846	LR583621	LR583731	LR583939		MW834144

Neocosmospora metavorans	CBS 143219 = NRRL 46708 = FMR 8634	Spain	Human foot	LR583851	LR583629	LR583744	LR583948		MW218081
Neocosmospora metavorans	CBS 135789	Greece, Athens	Pleural effusion of *Homo sapiens*	LR583849	LR583627	LR583738	LR583946		MW218080
Neocosmospora metavorans	**NFCCI 5193**	India, Jalgaon, Maharashtra	Soil	ON398191	ON032399	ON003504	OM837218		OM960758
Neocosmospora metavorans	**NFCCI 3475**	India, Amaravati, Maharashtra	*Ocimum sanctum* (endophyte)	ON398200	ON032408	ON003513	OM837227		OM960766
Neocosmospora metavorans	**NFCCI 4095**	India, Pune, Maharashtra	Soil	ON398202	ON032410	ON003515	OM837229		OM960768
Neocosmospora metavorans	**NFCCI 5199**	India, Jalgaon, Maharashtra	Soil	ON398214	ON032422	ON003527	OM837241		
Neocosmospora metavorans	**NFCCI 4885**	India, Jobner, Jaipur, Rajasthan	Cucumber twig and leaf	ON398250	ON032458	ON003563	OM837277		OM960806
Neocosmospora mori	ATCC 44934 = CBS 145467 = MAFF 238539 = NRRL 22230.	Japan, Miyazaki	Twigs of *Morus alba*	EU329499	AF178358	DQ094305	DQ236347		MW834149
Neocosmospora noneumartii	CBS 115658 = FRC S-0661	Israel, Palestine	*Solanum tuberosum*	MW446618	LR583630	LR583745	LR583949		MW218082
Neocosmospora obliquiseptata	MAFF 246845 = NRRL 62611	Australia, Queensland, Beerwah	Ambrosia beetle (*Euwallacea* sp.)	KC691637	KC691535	KC691576	KC691576		
Neocosmospora oblonga	CBS 130325 = CDC B-4701= NRRL 28008	USA	Eye of Homo sapiens	LR583853	LR583631	LR583746	LR583950		MW834154
Neocosmospora oblonga	**NFCCI 2150**	Arctic Tundra	Soil	ON398199	ON032407	ON003512	OM837226		OM960765
Neocosmospora oligoseptata	CBS 143241 = FRC S-2581 = MAFF 246283 = NRRL 62579	USA, Pennsylvania, Dauphin	Female ambrosia beetle (*Euwallacea validus*)	KC691627	KC691538	KC691566	KC691566		MW834155
Neocosmospora paraeumartii	BBA 62215 = CBS 487.76 = NRRL 13997	Argentina	Decaying stem base of *Solanum tuberosum*	LR583855	DQ247549	LR583747	LR583951		MW834156
Neocosmospora parceramosa	CBS 115695 = CPC 1246	South Africa	Soil	JX435249	JX435149	JX435199	JX435199		MW218083

Neocosmospora perseae	CBS 144142 = CPC 26829	Italy, Catania, San Leonardello	Trunk canker lesions on *Persea americana*	LT991909	LT991902	LT991940	LT991947		MW218084
Neocosmospora rectiphora	CBS 125726 = FRC S-1842	Sri Lanka	Dead tree	MW834028	JF433026	JF433043	JF433043		MW834165
Neocosmospora rectiphora	CBS 125727T = GJS 02-89 = FRC S-1831	Sri Lanka	Dead tree	LR583871	DQ247509	JF433034	JF433034		MW834166
Neocosmospora solani	CBS 140079ET = NRRL 66304 = GJS 09-1466 = FRC S-2364	Slovenia	*Solanum tuberosum*	KT313623	KT313611	KT313633	KT313633		MW218088
Neocosmospora solani	**NFCCI 2315**	India, Goa	Potato	ON398180	ON032388	ON003493	OM837207		OM960747
Neocosmospora suttoniana	CBS 143214T = NRRL 32858	USA	Human wound	EU329630	DQ247163	DQ094617	DQ236659		MW218092
Neocosmospora suttoniana	CBS 143224 = NRRL 54972	USA	Equine eye	KC808336	KC808197	MG189940	MG189925		MW218093
Neocosmospora suttoniana	**NFCCI 5190**	India, Maharashtra	Ginger	ON398176	ON032384	ON003489	OM837203		
Neocosmospora suttoniana	**NFCCI 2961**	India, Beed, Maharashtra	Pigeon pea root (Wilted)	ON398216	ON032424	ON003529	OM837243		OM960779
Neocosmospora suttoniana	**NFCCI 5210**	India, Simbal, Himachal Pradesh	Chilli fruit	ON398249	ON032457	ON003562	OM837276		OM960805
Neocosmospora suttoniana	**NFCCI 4830**	India, Madurai, Tamilnadu	Cotton rot	ON398254	ON032462	ON003567	OM837281		OM960809
Neocosmospora suttoniana	**NFCCI 5211**	India, Udaipur, Rajasthan	Damping-off affected Onion plants	ON398255	ON032463	ON003568	OM837282		
Neocosmospora vasinfecta	CBS 325.54 = ATCC 16238 = IFO 7591 = IMI 251386 = NRRL 22436	South Africa	Soil	JX171610	AF178348	AF178412	AF178381		
Neocosmospora vasinfecta	CBS 446.93 = IMI 316967 = NHL 2919	Japan	Soil	LR583898	LR583670	LR583791	LR583996		MW834175
Neocosmospora vasinfecta	CBS 533.65 = IMI 302625	India	Unknown	LR583899	LR583671	LR583792	LR583997		MW834176
Neocosmospora vasinfecta	ATCC 62199 = NRRL 22166	USA, Illinois	Cyst of *Heterodera glycines*	EU329497	AF178350	DQ094319	DQ236361		
Neocosmospora vasinfecta	**NFCCI 2960**	India, Amravati, Maharashtra	Pigeon pea root	ON398196	ON032404	ON003509	OM837223		OM960762

Neocosmospora vasinfecta	**NFCCI 2972**	India, Ahmednagar, Maharashtra	Pigeon pea (root)	ON398228	ON032436	ON003541	OM837255		OM960789

Isolates used in this study are in bol
